# Comparing the Efficacy and Adverse Events of Available COVID-19 Vaccines Through Randomized Controlled Trials: Updated Systematic Review and Network Meta-analysis

**DOI:** 10.34172/jrhs.2023.128

**Published:** 2023-12-29

**Authors:** Shima Hossaini, Fariba Keramat, Zahra Cheraghi, Bushra Zareie, Amin Doosti-Irani

**Affiliations:** ^1^Department of Epidemiology, School of Public Health, Hamadan University of Medical Sciences, Hamadan, Iran; ^2^Department of Infectious Disease, School of Medicine, Hamadan University of Medical Sciences, Hamadan, Iran; ^3^Modeling of Noncommunicable Diseases Research Center, Hamadan University of Medical Sciences, Hamedan, Iran; ^4^Research Center for Health Sciences, Hamadan University of Medical Sciences, Hamadan, Iran

**Keywords:** COVID-19 Vaccines, Vaccine efficacy, Network meta-analysis

## Abstract

**Background:** Different vaccines have so far been developed and approved to cope with COVID-19 in the world. The aim of this updated network meta-analysis (NMA) was to compare and rank all available vaccines in terms of efficacy and complications simultaneously.

**Study Design:** A systematic review.

**Methods:** Three major international databases, including Web of Science, Medline via PubMed, and Scopus, were searched through September 2023. The transitivity assumption was evaluated qualitatively in terms of epidemiologic effect modifiers. The exposure of interest in this study was receiving any available COVID-19 vaccine, and the primary outcome of interest was the incidence of symptomatic COVID-19. In this NMA, the relative risk of symptomatic COVID-19 was used to summarize the efficacy of vaccines in preventing COVID-19. The data were analyzed using the frequentist-based approach, and the results were reported using a random-effects model. Finally, the vaccines were ranked using a P-score.

**Results:** In total, 34 randomized controlled trials (RCTs) met the eligibility criteria for this systematic review and NMA out of 3682 retrieved references. Based on the results of the NMA, mRNA-1273 was the most effective vaccine in preventing COVID-19 and demonstrated the highest P-score (0.93). The relative risk (RR) for mRNA-1273 versus placebo was 0.07 (95% confidence interval [CI]: 0.03, 0.17). The second and third-ranked vaccines were BNT-162b2 (RR=0.08; 95% CI: 0.04, 0.15; P-score=0.93) and Gam-COVID-Vac (0.09; 95% CI: 0.03, 0.25; 0.88).

**Conclusion:** Based on the results of this NMA, it seems that all available vaccines were effective in COVID-19 prevention. However, the top three ranked vaccines were mRNA-1273, BNT-162b2, and Gam-COVID-Vac, respectively.

## Background

 In the COVID-19 pandemic, providing an effective vaccine was one of the main concerns of health policymakers and scientists. Consequently, different vaccines have been developed and approved to cope with this disease around the world. As of July 10, 2023, a total of almost 13.500 billion vaccine doses had been administered worldwide. As of July 19, 2023, the reported confirmed cases of COVID-19 and the deaths due to this disease were over nearly 750 million and nearly seven million, respectively.^1^ Although the COVID-19 pandemic has subsided, this disease still exists in the world, and there is a risk of future epidemics; thus, countries should be ready to combat it. Vaccination is one of the most effective strategies for preventing infectious diseases.

 The available vaccines include DNA, mRNA, vector, protein subunit, inactivated virus, live attenuated, and non-replicating viral vector vaccines.^2^ Although all available vaccines are effective in preventing COVID-19^3^ selecting the best vaccine among the available vaccines is a main challenge for health policymakers. The first approved vaccine against the COVID-19 virus was Pfizer. The efficacy of this vaccine in phase 3 randomized controlled trials (RCTs) with over 40,000 participants was 91.3%.^4^ After Pfizer, other countries and companies developed other vaccines. The Sinovac, AstraZeneca, Russian Sputnik, Johnson & Johnson, and Moderna vaccines have an efficacy of 51%,^5^ 63%,^6^ 97.6%,^7^ 66.9%,^8^ and 93.2%,^9^ respectively. In addition, the efficacy of Soberana 02 and Soberana Plus vaccines is 49.7 and 64.9%,^10^ respectively.

 In most phase 3 RCTs, all the vaccines have been compared with a placebo, so the safety, efficacy, and complications of the vaccines have been compared directly with a placebo, but a major question is regarding the simultaneous comparisons of all the available vaccines in terms of safety, efficacy, and complications two by two. It would be ideal if we had access to an RCT comparing all vaccines simultaneously, but there are no such RCTs. In the absence of such trials, indirect comparison via network meta-analysis (NMA) may be useful for simultaneous comparison.

 To date, there have been a few NMAs that have compared vaccines simultaneously. In an NMA that compared nine vaccines, BNT162b2, mRNA-1273, and Gam-COVID-Vac were the top three vaccines in terms of efficacy.^11^ Based on the results of a systematic review and NMA of 35 trials, the mRNA vaccines were most effective in preventing COVID-19.^3^ In an NMA comparing 16 vaccines for efficacy based on the results of this study, BNT126b2, mRNA-1273, and rAd26 & rAd5 vaccines were the top three vaccines.^12^ Based on the results of another NMA comparing 28 vaccines, the Pfizer vaccine was the most effective in preventing severe COVID-19 infection.^13^ Although there are some published NMAs,^12-14^ the vaccines included in these NMAs are not all ones that are available now because the results of some of the phase 3 trials have not been published. Accordingly, the aim of this updated NMA was to compare and rank all available vaccines with published results of phase 3 trials in terms of efficacy and complications simultaneously.

## Methods

 This NMA is part of a comprehensive systematic review that has simultaneously compared all available vaccines for safety, immunogenicity, efficacy, and related complications in phase 1, 2, and 3 RCTs. In this NMA, we analyzed only the results of phase 3 RCTs. In this systematic review and NMA, we followed the PRISMA guidelines for NMA.^15^ The efficacy of the vaccine is the performance of a vaccine under idealized conditions of an RCT.^16^

###  Search Strategy

 A search strategy was developed to identify all pertinent RCTs. Our search strategy is presented in Table S1 (see Supplementary file 1). Three major international databases, including Web of Science, Scopus, and Medline via PubMed, were searched through September 2023. We set up alerts in these databases and continued updating our search until the time of analysis.

###  Eligibility Criteria and Study Selection

 All phase 3 RCTs comparing COVID-19 vaccines with either a placebo or another vaccine were included regardless of study location, population, or language. The phase 1, 2, and 4 studies and non-randomized trials were excluded from this NMA.

 Two authors (Sh. H.) and (B. Z.) were responsible for screening the results of our search. All retrieved studies were imported into EndNote software (version X7), and duplicate studies were identified by software and manual review and finally excluded from the pool of studies. Next, the two authors mentioned above independently screened the studies based on their titles and abstracts. Any disagreement between the two authors was resolved by discussion and the judgment of the third reviewer (A. D. I.). Finally, the full texts of selected RCTs were screened according to the mentioned inclusion criteria, and eligible RCTs were identified for data extraction.

 The study’s primary and secondary outcomes included the frequency of symptomatic COVID-19 infection and vaccine complications such as localized reactions, fatigue, chills, fever, pain, and headache.

###  Data Extraction

 The eligible RCTs were analyzed, with data extracted on the characteristics of the RCTs, such as the first author’s name, publication year, country, study population, duration of follow-up, data-analysis approach (intention to treat or per protocol), and sample size; the other obtained data were vaccine data (i.e., the exact type of vaccine used in each RCT), potential effect modifiers (e.g., gender and age of participants), and outcomes (i.e., the number of confirmed COVID-19 cases in the vaccine and placebo groups, and efficacy with a 95% confidence interval [CI]), and any reported adverse events in the vaccine and placebo groups.

###  Risk of Bias Assessment

 The Cochrane tool was used to assess the risk of bias.^17^ Two authors (Sh. H. and A. D. I.) were responsible for the risk of bias assessment. Several items from this tool were used, including random sequence generation, allocation concealment, blinding of participants and personnel, blinding of outcome assessment, incomplete outcome data, and selective reporting. The included RCTs were classified as low, high, moderate, and risk of bias if all items were met, if one item was not met, and if more than one item was not met, respectively.^17^ Review Manager 5.4 was utilized to assess the risk of bias.^18^

###  Data Analysis

 The transitivity assumption was evaluated qualitatively in terms of epidemiologic effect modifiers. In this NMA, age and the study population were considered the main effect modifiers. The heterogeneity of pairwise comparisons and the network was assessed using the χ^2^ test and the I^2^ statistic. The restricted maximum likelihood estimator was used to calculate the between-study variance.^19^ The consistency assumption was not assessed in this NMA because there was no closed loop in our networks.^20^ The available vaccines were presented through a network diagram. The study employed relative risk (RR) to summarize their efficacy in preventing COVID-19 in the NMA. The obtained data were analyzed using the frequentist-based approach, and the results were reported by a random-effects model.

 Eventually, the vaccines were ranked using a P-score. The value of the P-score is between zero and one, and a higher value of the P-score indicates a better rank for a vaccine. The P-score for each vaccine is calculated using the one-sided *P*-value of rejecting the null hypothesis (Pj). In a network, the P-score for each treatment is the mean of all 1-P[j].^21^ Publication bias was evaluated visually using an adjusted network funnel plot and Egger test.^22^ The results were reported with a 95% CI. Statistical analysis was conducted using R version 4.0.0 (2020-04-24), and the “netmeta” package was used for NMA.

## Results

**Figure 1:**
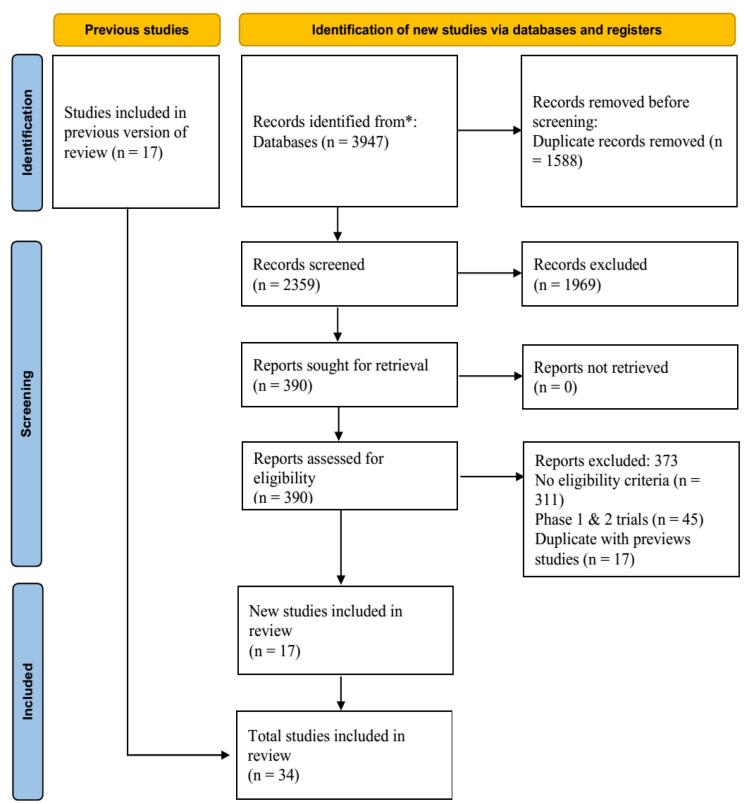
The flow diagram for the process of study identification for network meta-analysis

**Figure 2:**
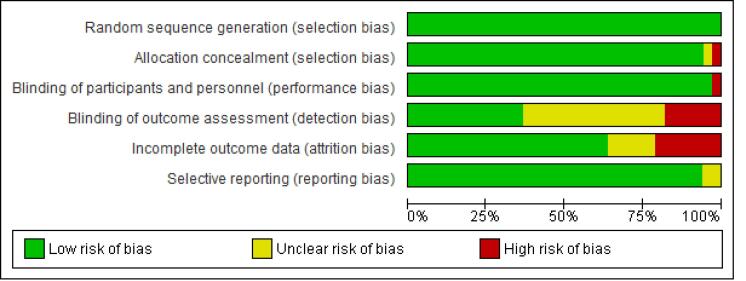
Risk of bias graph: review authors’ judgements about each risk of bias item presented as percentages across all included studies

 Overall, 34 RCTs^4-7,9,10,23-50^ met the eligibility criteria for this systematic review and NMA out of 3682 retrieved references (Figure 1). Of these studies, 26, 5, and 2 RCTs were conducted only on adults of both genders, only on children, on people aged 50 years and older, respectively, and one study was performed on both adults and children. Based on our assessment of the transitivity assumption, the included RCTs were divided into those conducted on adults, children, and the elderly. The results of the risk of bias assessment are shown in Figure 2. The characteristics of the included RCTs are provided in Table 1.

**Table 1:** Characteristics of the included randomized controlled trials in the study

**Author (y)**	**Country**	**Study population**	**Sample size**	**Mean age (y)**	**Male proportion (%)**	**Median duration of follow-up (days)**	**Analysis**	**Loss to Follow-up (%)**	**Vaccines/Placebo**	**Confirmed Cases of COVID-19**	**Efficacy (95% CI)**
Polack (2020) ^4^	International	Healthy adults	total: 43548 n1: 18860 n2: 18846	Arm1: 52 Arm2: 52	Arm1:51.1 Arm2: 50.1	60	ITT	Arm1: 1.6 Arm2: 1.7	Arm1: BNT162b2(30µg) Arm2: Placebo	Arm1: 9 Arm2: 169	94.6 (89.9, 97.3)
Tanriover (2021) ^5^	Turkey	Healthy adults	total: 10218 n1: 6650 n2: 3568	Arm1: 45 Arm2: 45	Arm1:57.4 Arm2:	43	ITT	Arm1: 1.4 Arm2: 2.7	Arm1: CoronaVac Arm2: Placebo	Arm1:9 Arm2: 32	83.5 (65.4, 92.1)
Emary (2021) ^6^	UK	Healthy adults	total: 8534 n1: 4244 n2: 4290	Arm1: 45 Arm2: 45	Arm1:41.4 Arm2: 39.9	48	Interim analysis	Arm1: 32.0 Arm2: 21.5	Arm1: ChAdOx1 nCoV-19 Arm2: MenACWY	Arm1:59 Arm2: 210	72.3 (63.1, 79.3)
Logunov (2021) ^7^	Russia	Healthy adults	total: 21977 n1: 16501 n2: 5476	Arm1: 45.3 Arm2: 45.3	Arm1:55.4 Arm2: 55.1	48	PP	Arm1: 0.4 Arm2: 0.7	Arm1: Gam-COVID-Vac Arm2: Placebo	Arm1:13 Arm2: 47	91.1 (83.8, 95.1)
Baden (2021) ^9^	USA	Healthy adults	total: 30415 n1: 15209 n2: 15206	Arm1: 51.4 Arm2: 51.3	Arm1:52.1 Arm2: 53.0	60	ITT*	Arm1: 3.0 Arm2: 3.0	Arm1: mRNA-1273(100µg) Arm2: Placebo	Arm1:55 Arm2: 751	93.2 (91.1, 94.9)
Mostafavi (2023) ^10^	Iran	Healthy adults	total: 18000 n1: 14375 n2: 3597	Arm1: 39.4 Arm2: 39.1	Arm1:60.1 Arm2: 59.1	100	ITT	Arm1: 3.7 Arm2: 4.0	Arm1: FINLAY-FR-2 (25 μg) Arm2: Placebo	Arm1:461 Arm2: 221	49.7 (40.8, 57.3)
Mostafavi (2023) ^10^	Iran	Healthy adults	total: 6000 n1: 4790 n2: 1197	Arm1: 39.6 Arm2: 39.9	Arm1:59.8 Arm2: 59.9	142	ITT	Arm1: 12.8 Arm2: 13.2	Arm1: FINLAY-FR-2(25 μg) + FINLAY-FR-1A (50 μg) Arm2: Placebo	Arm1:75 Arm2: 51	64.9 (49.7, 59.5)
Kaabi (2021)^23^	UAE	Healthy adults	total: 40411 n1: 13066 n2: 13068 n3: 13071	Arm1: 36.2 Arm2: 36.1 Arm3: 36.1	Arm1: 81.9 Arm2: 82.3 Arm3: 82.7	77	PP*	Arm1: 3.0 Arm2: 2.9 Arm2: 3.0	Arm1: SARS-CoV-2 WIV04 (0.5 µg) Arm2: HB02 (4 µg) Arm3: Alum	Arm1: 26 Arm2: 21 Arm3: 95	72.8 (58.1, 82.4) 78.1 (64.8, 86.3)
Ali (2021) ^24^	USA	Young adults	total: 3732 n1: 2489 n2: 1243	Arm1: 14.3 Arm2: 14.2	Arm1:51.5 Arm2: 50.8	83	PP	Arm1: 2.5 Arm2: 16.6	Arm1: mRNA-1273 (100 µg) Arm2: Placebo	Arm1:1 Arm2: 7	93.3 (47.9, 99.9)
Bravo (2022) ^25^	Belgium & …	Healthy adults	total: 30174 n1: 15092 n2: 15082	Arm1: 31.2 Arm2: 31	Arm1:22.5 Arm2: 22.5	60	PP	Arm1: 58.4 Arm2: 59.4	Arm1: SCB-2019 (30 µg) Arm2: Placebo	Arm1:63 Arm2: 185	67.2 (54.3, 76.8)
C.B. Creech (2022) ^26^	USA	Children	total: 4016 n1: 3012 n2: 1004	Arm1: 8.5 Arm2: 8.5	Arm1:51.6 Arm2: 47.9	82	PP	Arm1: 1.6 Arm2: 14.6	Arm1: mRNA-1273 (50 µg) Arm2: Placebo	Arm1:3 Arm2: 4	88.0 (70.0, 95.8)
Dai (2020) ^27^	Asia	Healthy adults	total: 28904 n1: 14453 n2: 14451	Arm1: 52 Arm2: 52	Arm1:51.1 Arm2: 50.1	50.4	ITT	Arm1: 1.6 Arm2: 1.7	Arm1: ZF2001 Arm2: Placebo	Arm1: 36 Arm2: 188	81.4 (73.3, 87.3)
L.M. Dunkle (2022) ^28^	USA	Healthy adults	total: 29949 n1: 19965 n2: 9984	Arm1: 47 Arm2: 47	Arm1:45.3 Arm2: 41.4	60	PP	Arm1: 13.3 Arm2: 18.5	Arm1: NVX-CoV2373 Arm2: Placebo	Arm1:14 Arm2: 63	90.4 (82.9, 94.6)
Ella (2021) ^29^	India	Healthy adults	total: 25798 n1: 12899 n2: 12899	Arm1: 40.1 Arm2: 40.1	Arm1:67.2 Arm2: 66.8	146	PP	Arm1: 6.9 Arm2: 6.9	Arm1: BBV152 Arm2: Placebo	Arm1: 24 Arm2: 106	77.8 (65.2, 86.4)
Fadlyana (2021) ^30^	Indonesia	Healthy adults	total: 1819 n1: 811 n2: 809	Arm1: 35.6 Arm2: 35.4	Arm1:62.3 Arm2: 66.9	90	ITT	Arm1: 1.5 Arm2: 0.7	Arm1: Sinovac Arm2: Placebo	Arm1:7 Arm2: 18	65.3
Falsey (2021) ^31^	USA	Healthy adults	total: 63171 n1: 42352 n2: 20747	Arm1: 50.2 Arm2: 50.2	Arm1:28.4 Arm2: 28.9	61	ITT	Arm1: 1.5 Arm2: 2.4	Arm1: ChAdOx1 nCoV-19 Arm2: Placebo	Arm1:168 Arm2: 214	74.0 (65.5, 80.5)
Frenck Jr. (2021) ^32^	USA	Healthy adults	total: 2264 n1: 1134 n2: 1130	Arm1: 13.6 Arm2: 13.6	Arm1:50.0 Arm2: 51.8	60	ITT	Arm1: 1.4 Arm2: 2.5	Arm1: BNT162b2 Arm2: Placebo	Arm1:0 Arm2: 16	100 (75.3, 100.0)
Frenck Jr. (2021) ^32^	USA	Children	total: 3788 n1: 1875 n2: 1913	Arm1: 19.4 Arm2: 19.6	Arm1:13.6 Arm2: 14.1	60	ITT	Arm1: 3.8 Arm2: 5.5	Arm1: BNT162b2 Arm2: Placebo	Arm1: NR Arm2: NR	100.0
B. Gilbert (2022) ^33^	USA	Healthy adults	total: 1147 n1: 1010 n2: 137	Arm1: NR Arm2: NR	Arm1:NR Arm2: NR	116	PP	Arm1: NR Arm2: NR	Arm1: mRNA-1273 Arm2: Placebo	Arm1:NR Arm2: NR	78.0 (54.0, 89.0)
Halperin (2022) ^34^	Chile & …	Healthy adults	total 36982 n1: 18493 n2: 18489	Arm1: 37.8 Arm2: 37.7	Arm1:40.3 Arm2: 41.0	45	ITT	Arm1: 2.6 Arm2: 2.5	Arm1: Ad5-nCoV Arm2: Placebo	Arm1:45 Arm2: 105	57.5 (39.7, 70.0)
Hardt (2022) ^35^	International	Healthy adults	total: 31300 n1: 15708 n2: 15592	Arm1: 53 Arm2: 53	Arm1:52.9 Arm2: 52.3	70	PP	Arm1: 52.4 Arm2: 55.1	Arm1: Ad26.COV2.S Arm2: Placebo	Arm1:14 Arm2: 53	75.6 (55.5, 87.5)
Heath (2023) ^36^	United Kingdom	Healthy adults	total: 15185 n1: 7569 n2: 7569	Arm1: 53.4 Arm2: 53.4	Arm1:47.5 Arm2: 47.8	135	ITT	Arm1: 0.0 Arm2: 0.0	Arm1:NVX-CoV2373 Arm2: Placebo	Arm1:134 Arm2: 24	68.7 (58.1, 76.6)
Khairullin (2022) ^37^	Kazakhstan	Healthy adults	total: 3000 n1: 2400 n2: 600	Arm1: 35 Arm2: 34	Arm1:50.2 Arm2: 52.2	180	ITT	Arm1: 3.0 Arm2: 2.7	Arm1:QazCovid-in(5µg) Arm2: Placebo	Arm1:31 Arm2: 43	82 (71.1, 88.5)
Khobragade (2022) ^38^	India	Healthy adults	total: 27703 n1: 13851 n2: 13852	Arm1: 36.4 Arm2: 36.6	Arm1:67.5 Arm2:	350	PP	Arm1: 1.5 Arm2: 1.6	Arm1:ZyCoV-D(2mg) Arm2: Placebo	Arm1:20 Arm2: 61	66.6 (47.6, 80.7)
Kremsner (2022) ^39^	10 countries	Healthy adults	total: 39680 n1: 19846 n2: 19834	Arm1: 43 Arm2: 43	Arm1:54.7 Arm2: 54.5	48.2	ITT	Arm1: 2.1 Arm2: 6.8	Arm1:CVnCoV Arm2: Placebo	Arm1:83 Arm2: 145	70.7(42.5, 86.1)
Lioznov (2023) ^40^	Russia	Healthy adults	total: 783 n1: 374 n2: 126	Arm1: 41.2 Arm2: 41	Arm1:40.4 Arm2: 38.1	210	PP	Arm1: 3.5 Arm2: 4.8	Arm1: Ad5-nCoV(0·5mL) Arm2: Placebo	Arm1:18 Arm2: 13	NR
Moreira (2022) ^41^	USA	Healthy adults	total:10136 n1: 5088 n2: 5048	Arm1: 51.8 Arm2: 51.7	Arm1:48.3 Arm2: 49.9	75	interim analysis	Arm1: 0.2 Arm2: 0.9	Arm1:BNT162b2(30µg) Arm2: Placebo	Arm1:15 Arm2: 141	89.8 (82.6, 94.4)
F.M. Muñoz (2023) ^42^		Children 6 Months to < 2 Y	total: 1776 n1: 1178 n2: 598	Arm1: 1.26 Arm2: 1.28	Arm1:50 Arm2:	40	ITT	Arm1: 0.8 Arm2: 0.7	Arm1:BNT162b2(3µg) Arm2: Placebo	Arm1:4 Arm2: 8	75.8 (9.7, 94.7)
F.M. Muñoz (2023) ^42^		Children 2 to 4 Yr	total: 2750 n1: 1835 n2: 915	Arm1: 3 Arm2:	Arm1:49.1 Arm2: 51.5	42	ITT	Arm1: 1.3 Arm2: 2.6	Arm1:BNT162b2(3µg) Arm2: Placebo	Arm1:9 Arm2: 13	71.8 (28.6, 89.4)
Sadoff (2022) ^43^	USA	Healthy adults	total: 43788 n1: 21898 n2: 21890	Arm1: 52 Arm2: 52	Arm1:55.1 Arm2:	120	PP	Arm1: 10.6 Arm2: 10.4	Arm1:Ad26.COV2.S Arm2: Placebo	Arm1:433 Arm2: 883	52.9 (47.1, 58.1)
Sobieszczyk (2022) ^44^	USA	Healthy adults	total: 32450 n1: 21634 n2: 10816	Arm1: 51 Arm2: 51	Arm1:55.5 Arm2: 55.5	78	interim analysis	Arm1: 9.5 Arm2: 18.0	Arm1:AZD1222 Arm2: Placebo	Arm1:335 Arm2: 224	67.0 (58.9, 73.4)
S. J. Thomas (2021) ^45^	USA	Healthy adults	total: 44165 n1: 22085 n2: 22080	Arm1: 51 Arm2: 51	Arm1:51.3 Arm2: 50.0	180	PP	Arm1: 1.5 Arm2:	Arm1:BNT162b2(30µg) Arm2: Placebo	Arm1:3 Arm2: 35	91.3 (89.0, 93.2)
Toback (2021) ^46^	UK	Healthy adults	total: 15187 n1: 217 n2: 214 n3:502 n4:497	Arm1: 42.3 Arm2: 41.9 Arm2: 51.6 Arm2: 51.4	Arm1:56.7 Arm2: 55.1 Arm2: 51.4 Arm2: 58.4	60	ITT	Arm1: 0.0 Arm2: 0.0 Arm3: 0.0 Arm3: 0.0	Arm1:NVX-CoV2373(5µg) + influenza Arm2: Placebo + influenza Arm2: NVX-CoV2373(5µg) Arm2: Placebo	Arm1:2 Arm2: 8 Arm3: 1 Arm4: 8	87.5 (0.2, 98.4)
Torales (2022) ^47^	Paraguay	Healthy adults	total: 1105 n1: 520 n2: 510	Arm1: 32.1 Arm2: 32.2	Arm1:58.5 Arm2: 61.8	28	interim analysis	Arm1: 10.4 Arm2: 10.0	Arm1:MVC-COV1901 Arm2: AZD1222	Arm1: NR Arm2: NR	62.6 (50.9, 71.5)
Walter (2022) ^48^	USA	Children	total: 2285 n1: 1528 n2: 757	Arm1: 8.2 Arm2: 8.1	Arm1:52.3 Arm2: 50.6	69	ITT	Arm1: 1.2 Arm2: 1.5	Arm1:BNT162b2(30µg) Arm2: Placebo	Arm1:3 Arm2: 16	90.7 (67.4, 98.3)
Winokur (2022) ^49^	USA	Healthy adults	total: 1846 n1: 306 n2: 302 n3: 308 n4: 308 n5: 306 n6: 316	Arm1: 66 Arm2: 67 Arm2: 67 Arm2: 67 Arm2: 67 Arm2: 67	Arm1:47.4 Arm2: 48.0 Arm2: 50.0 Arm2: 49.7 Arm2: 52.9 Arm2: 48.4	51	ITT	Arm1: 1.3 Arm2: 0.3 Arm3: 1.0 Arm4: 1.3 Arm5: 0.7 Arm6: 0.9	Arm1:BNT162b2(30µg) Arm2: BNT162b2(60µg) Arm3: monovalent BA.1(30µg) Arm4: monovalent BA.1(60µg) Arm5: bivalent BA.1(30µg) Arm6: bivalent BA.1(60µg)	Arm1:7 Arm2: 6 Arm3: 7 Arm4: 3 Arm5: 1 Arm6: 6	NR
Mohraz (2023) ^50^	Iran	Healthy adults	total: 20000 n1: 13335 n2: 6665	Arm1: 38.3 Arm2: 38.2	Arm1: 65.5 Arm2: 65.4	83	PP	Arm1: 0.49 Arm2: 0.23	Arm1: BIV1-CovIran Arm2: Placebo	Arm1: 758 Arm2: 688	50.2 (44.7, 55.0)

*Note*. PP: Per-protocol; ITT: Intention to treat; NR: Not reported; CI: Confidence interval.

**Figure 3:**
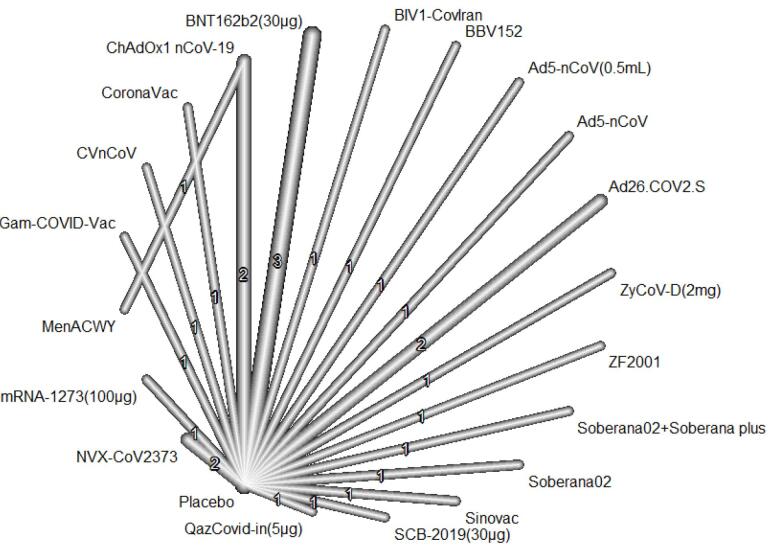
The network plot of the available COVID-19 vaccines compared in phase three trials

 The incidence of confirmed cases of symptomatic COVID-19 among adults has been reported in 25 RCTs. These RCTs formed two subnetworks involving 23 vaccines and 20 designs. The first subnetwork entails 24 RCTs with 24 pairwise comparisons, 20 vaccines, one placebo, and 19 designs. Figure 3 illustrates the visual presentation of this network. The I2 value for this network was 84.7%, and the p-value for heterogeneity testing (within the design) was < 0.001. There was no indication of publication bias in this NMA, as the *P* value of the Egger test was 0.308.

 The efficacy of vaccines has been reported in 27 RCTs. The highest reported efficacy (99.0%; 95% CI: 75.0, 100.0) was associated with BNT162b2 (30 µg), according to Table 1.

**Figure 4:**
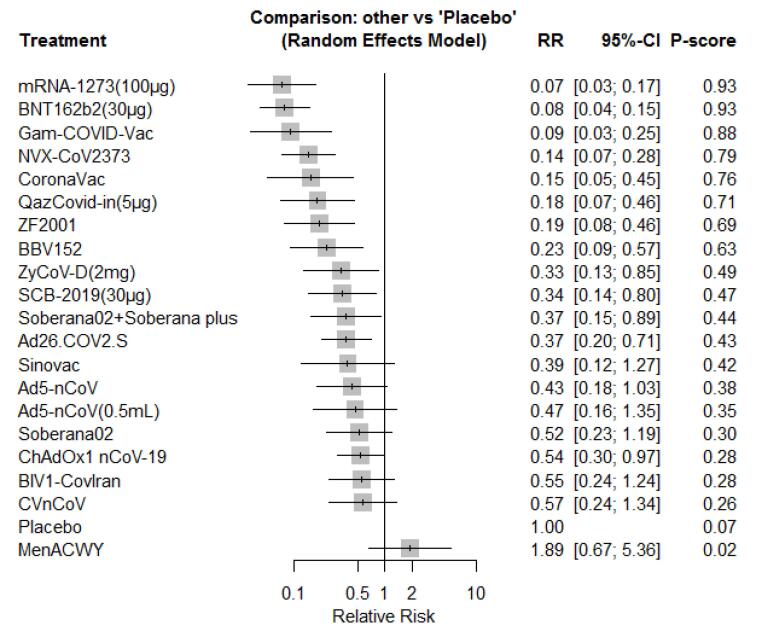
The forest plot for the comparison of the available COVID-19 vaccines versus placebo in the network meta-analysis

 Based on the results of the NMA and the simultaneous comparison of all vaccines versus placebo, mRNA-1273 was the most effective vaccine in preventing COVID-19, and the highest P-score (0.93) was associated with this vaccine. The RR for mRNA-1273 versus placebo was 0.07 (95% CI: 0.03, 0.17). The second- and third-ranked vaccines were BNT-162b2 (RR = 0.08; 95% CI: 0.04, 0.15; P-score = 0.93) and Gam-COVID-Vac (RR = 0.09; 95% CI: 0.03, 0.25; P-score = 0.88). Overall, all vaccines, except for MenACWY, were significantly effective in preventing COVID-19 (Figure 4). The pooled comparisons of all vaccines are presented in Table S2 (see Supplementary file 1). The vaccines in a three-arm RCT were not connected to the network.^24^ In this study, two inactivated vaccines, including SARS-CoV-2 WIV04 and HB02, were compared with aluminum hydroxide. Based on the results of this study, the vaccine efficacy for WIV04 and HB02 was 72.8% and 78.1%, respectively.

 The included RCTs evaluating the vaccines in children were five RCTs^24,26,32,42,48^ with six pairwise comparisons, five interventions, and four designs. The visual representation of the vaccine network is depicted in Figure S1(see Supplementary file 1). The I^2^ value for this network and the *P* value for the test of heterogeneity (within design) were 0 and 0.710, respectively. In children, the highest efficacy was associated with BNT162b2 (30 µg, 99.0%; 95% CI: 75.3, 100.0) in an RCT by Frenck et al^32^ (Table 1).

 Based on the results of the NMA, BNT162b2 (30 µg) was the most effective vaccine in children (P-score = 0.84). The RR for comparing BNT162b2 (30 µg) with a placebo was 0.08 (95% CI: 0.03, 0.24). Overall, all vaccines were effective in preventing COVID-19 in this group compared to placebo Figure S2 (see Supplementary file 1). The pooled comparisons of all vaccines in this group are provided in Table S3 (see Supplementary file 1).

 Two RCTs were conducted on people over 55 years of age. In the study by Sadoff et al comparing single-dose Ad26.CoV2.S with a placebo, vaccine efficacy ≥ 14 days and ≥ 28 days after administration was 55.0% (95% CI: 42.9, 64.7) and 46.6% (95% CI: 30.7, 59.0), respectively.^43^ In another RCT by Winokur et al, BNT162b2 (30 µg), BNT162b2 (60 µg), monovalent BA.1 (30 µg), monovalent BA.1 (60 µg), bivalent BA.1 (30 µg), and bivalent BA.1 (60 µg) were compared, there was no significant difference among the mentioned vaccines in terms of the incidence of confirmed cases of COVID-19 after administration.^49^

Table 2 summarizes the RR for the incidence of major vaccine complications, including local reactions, fatigue, chills, fever, pain, and headache. Based on the results of NMA for the mentioned complications, the risk of local reaction for Ad5-nCoV (0.5 mL) was the highest compared with a placebo among RCTs reporting this complication. The risks of fatigue, chills, fever, pain, and headache were the highest for Sinovac, BNT162b2, BNT162b2, Sinovac, and BNT162b2 (30 µg), respectively. Among children, BNT162b2 was associated with the highest risk of the above-mentioned complications. The simultaneous comparisons of the vaccines for the incidence of local reactions, fatigue, chills, fever, pain, and headache are listed in Tables S4-S9(see Supplementary file 1).

**Table 2:** The relative risk for the complications of each vaccine versus the placebo

**Vaccines**	**Local Reaction**	**Fatigue**	**Chill**	**Fever**	**Pain**	**Headache**
Ad26.CoV2.S	3.04 (2.51, 3.67)	1.89 (1.48, 2.42)	Unreported	Unreported	2.69 (2.08, 3.47)	1.84 (1.49, 2.27)
Ad5-nCoV	3.09 (2.33, 4.10)	1.68 (0.56, 5.09)	Unreported	7.92 (1.46, 42.83)	2.13 (1.47, 3.07)	1.45 (1.07, 1.97)
Ad5-nCoV (0·5 mL)	17.86 (4.37, 72.92)	Unreported	Unreported	3.16 (0.53, 18.73)	0.34 (0.17, 0.69)	1.24 (0.49, 3.11)
BIV1-CovIran	1.08 (0.83, 1.39)	3.37 (2.73, 4.15)	Unreported	Unreported	Unreported	Unreported
BNT162b2 (30 µg)	7.36 (6.24, 8.68)	1.67 (1.19, 2.35)	11.05 (7.31, 16.71)	Unreported	5.34 (4.24, 6.74)	2.88 (2.40, 3.46)
ChAdOx1 nCoV-19	2.5 (1.74, 3.59)	1.19 (0.83, 1.70)	3.96 (2.10, 7.48)	Unreported	3.59 (2.50, 5.17)	1.77 (1.33, 2.36)
CoronaVac	1.75 (1.20, 2.56)	2.71 (1.91, 3.84)	1.28 (0.64, 2.54)	1.22 (0.23, 6.47)	1.32 (0.89, 1.97)	0.96 (0.70, 1.32)
CVnCoV	5.54 (4.18, 7.35)	Unreported	9.89 (5.02, 19.46)	92.34 (14.40, 592.22)	5.91 (4.04, 8.65)	3.07 (2.27, 4.15)
Gam-COVID-Vac	0.55(0.33,0.90)	Unreported	Unreported	1.33 (0.09, 20.45)	0.83 (0.16, 4.43)	1.11 (0.50, 2.45)
mRNA-1273 (100 µg)	4.72 (3.66, 6.09)	2.81 (2.01, 3.93)	7.99 (4.22, 15.13)	52.92 (10.03, 279.34)	4.68 (3.31, 6.61)	2.52 (1.90, 3.34)
MVC-CoV1901	2.6 (1.59, 4.26)	1.93 (1.05, 3.55)	5.44 (1.79, 16.49)	Unreported	4.7 (2.48, 8.89)	1.77 (1.05, 2.99)
NVX-CoV2373	3.76 (2.91, 4.86)	2.34 (1.67, 3.28)	Unreported	21.16 (3.91, 114.36)	4.12 (2.91, 5.84)	2.34 (1.76, 3.12)
Placebo	1.00	1.00	1.00	1.00	1.00	1.00
QazCOVID-in (5 µg)	3.75 (2.25, 6.25)	0.25 (0.03, 1.82)	0.38 (0.09, 1.54)	0.47 (0.07, 2.97)	0.31 (0.08, 1.21)	0.75 (0.39, 1.45)
SCB-2019 (30 µg)	3.47 (2.36, 5.11)	1.35 (0.86, 2.12)	1.92 (0.75, 4.92)	2.50 (0.25, 25.33)	1.20 (0.71, 2.04)	1.10 (0.74, 1.64)
Sinovac	3.48 (2.19, 5.52)	6.27 (2.66, 14.81)	Unreported	2.00 (0.19, 21.04)	8.98 (4.03, 20.00)	Unreported
Soberana02	2.49 (1.92, 3.24)	1.2 (0.85, 1.70)	1.05 (0.52, 2.13)	1.16 (0.22, 5.99)	1.29 (0.87, 1.91)	1.07 (0.79, 1.44)
Soberana02 + Soberana plus	2.66 (1.93, 3.66)	1.21 (0.79, 1.85)	1.25 (0.36, 4.34)	1.64 (0.30, 8.92)	2.39 (1.18, 4.84)	1.11 (0.74, 1.67)
ZyCoV-D (2s mg)	1.06 (0.62, 1.82)	0.65 (0.28, 1.48)	Unreported	1.23 (0.21, 7.39)	1.17 (0.50, 2.71)	0.95 (0.49, 1.85)

## Discussion

 In this NMA, the available vaccines (20 vaccines versus a placebo) were ranked for the prevention of symptomatic COVID-19. Based on the results of this study, mRNA-1273, BNT162b2, and Gam-COVID-Vac were the most effective vaccines in adults. In children, BNT162b2 was the most effective vaccine. Overall, all vaccines, except for MenACWY, were significantly effective in preventing COVID-19 in adults. Local reactions, fatigue, chills, fever, pain, and headaches were the common complications in the included RCTs. The risk of these complications was the highest for Ad5-nCoV (0.5 mL), Sinovac, BNT162b2 (30 µg), BNT162b2 (30 µg), Sinovac, and BNT162b2 (30 µg) versus a placebo, respectively. In this NMA, the previously published NMAs were updated, and the latest published RCTs were included in this study.

 In a published NMA of nine vaccines, BNT162b2 mRNA-1273, followed by Gam-COVID-Vac, were ranked with the highest probability of efficacy against symptomatic COVID-19.^11^ Our results are in line with a published NMA in 2022, showing that BNT162b2, mRNA-1273, and rAd26&rAd5 (Gam-COVID-Vac) were the three best vaccines, respectively.^12^ The results of a previously published NMA from 2021 aligned with our findings concerning symptomatic COVID-19 prevention.^13^ According to this NMA, Pfizer, Moderna, and Sputnik were the most effective vaccines, which is consistent with our results. Our study added value to the previous NMA by simultaneously comparing 20 vaccines. Overall, our findings confirmed those of prior NMA studies.

 In this study, comparing different doses of BNT162b2 and mRNA-1273 mRNA vaccines in children and adolescents, all doses were effective in preventing symptomatic COVID-19. However, BNT162b2 (30 μg) was found to be the most effective vaccine. These findings align with other published NMAs, suggesting that mRNA vaccines are the most effective in preventing symptomatic COVID-19. Despite opposition from some companies regarding the use of mRNA-based vaccines,^51^ it appears that these platforms are effective in fighting the pandemic. Unlike protein-based vaccines that primarily stimulate antibody production, mRNA vaccines elicit both cellular and hormonal immune responses.^52^

 In addition to vaccine efficacy and disease prevention, the safety and incidence of complications are crucial considerations in vaccine use. The included RCTs reported varying complication profiles. To address this issue, our NMA analyzed the risk of commonly reported complications such as local reactions, fatigue, chills, fever, pain, and headaches. Based on our findings, the highest risk for local reactions, fatigue, chills, fever, pain, and headaches occurred for Ad5-nCoV (0.5 mL), as well as for the Sinovac and BNT162b2 vaccines. According to an NMA, Pfizer, QazCOVID-in, and Clover vaccines have the highest risk for local side effects. In terms of systemic side effects, the ZyCoV-D, V591, V-01, and Sinopharm vaccines were the safest options, while the Pfizer, Clover, and QazCOVID-in vaccines carried the highest risk of developing such effects.^13^ Vaccines, similar to any other medical intervention, come with potential complications. While common complications are identified in phases two and three of trials, the identification of rare complications requires phase four studies in post-licensing evaluations. Overall, the decision to introduce a new vaccine depends on the burden of the disease, vaccine efficacy and effectiveness, vaccine safety, and the costs and cost-effectiveness of the vaccine.^16^

 The key advantage of this study over previous NMAs is its comparison of multiple vaccines. For instance, the NMA includes findings from RCTs conducted in Iran on Soberana 02, Soberana Plus,^10^ and BIV1-CovIran vaccines.^50^

 We were unable to assess the consistency assumption in this NMA due to the absence of a closed loop in the vaccine network and the use of solely indirect estimates in the comparison of vaccines. Therefore, we could only evaluate the transitivity assumption qualitatively. Based on our evaluation of the transitivity assumption, we decided to conduct a subgroup NMA, including participants in different age groups [children and adolescents ( < 18 years old), adults (18-55 years old), and older adults ( > 55 years old)]. In this NMA, the available vaccines were ranked based on their ability to prevent symptomatic COVID-19. However, it is important to note that several factors, such as the virus strain, mutations, variations in the study population and setting, and the quality of the studies, were not accounted for in this NMA. Therefore, the results should be interpreted with caution.

**Highlights**

- The twenty-three available COVID-19 vaccines were compared and ranked simultaneously.
- All available vaccines are effective in preventing symptomatic COVID-19.
- MRNA-1273 (Moderna) was the most effective vaccine for preventing COVID-19.

## Conclusion

 Based on the NMA results, all available vaccines have proven effective in preventing COVID-19. However, the top three ranked vaccines were mRNA-1273, BNT-162b2, and Gam-COVID-Vac, with the mRNA vaccines taking the lead. It is important to note that BNT-162b2 has a high risk of complications, including fatigue, chills, fever, pain, and headaches.

## Acknowledgements

 This study was part of the MSc thesis in epidemiology. We would like to thank the Health Sciences Research Center and the Vice-chancellor for Research and Technology of the Hamadan University of Medical Sciences for supporting this study.

## Authors’ Contribution

**Conceptualization:** Amin Doosti-Irani.

**Data curation:** Shima Hossaini, Bushra Zareie, Amin Doosti-Irani.

**Formal analysis:** Amin Doosti-Irani and Shima Hossaini.

**Investigation:** Shima Hossaini, Fariba Keramat, Amin Doosti-Irani, Zahra Cheraghi.

**Methodology:** Amin Doosti-Irani, Shima Hossaini, Zahra Cheraghi.

**Project administration:** Amin Doosti-Irani.

**Software:** Amin Doosti-Irani, Shima Hossaini.

**Supervision:** Amin Doosti-Irani, Fariba Keramat.

**Validation:** Fariba Keramat, Zahra Cheraghi, Bushra Zarei, Amin Doosti-Irani.

**Visualization:** Amin Doosti-Irani, Shima Hossaini, Bushra Zareie.

**Writing–original draft:** Amin Doosti-Irani and Shima Hossaini.

**Writing–review & editing:** Amin Doosti-Irani, Zahra Cheraghi, Fariba Keramat, Shima Hossaini, Bushra Zareie.

## Competing Interests

 None.

## Funding

 None.

## Supplementary Files

Supplementary file 1 contains Figure S1 and Tables S1-S9.
