# Supplementary material for: Comparing the Efficacy and Adverse Events of Available COVID-19 Vaccines Through Randomized Controlled Trials: Updated Systematic Review and Network Meta-analysis
Source: J Res Health Sci. 2023 Dec 29;23(4):e00593. doi: 10.34172/jrhs.2023.128 (PMC10843317; doi:10.34172/jrhs.2023.128)
Supplement: Supplementary file 1 — contains Figure S1 and Tables S1-S9. [file jrhs-23-e00593-s001.pdf]

doi:10.34172/jrhs.2023.128
JRHS 2023; 23(4):e00593

Supplementary file 1

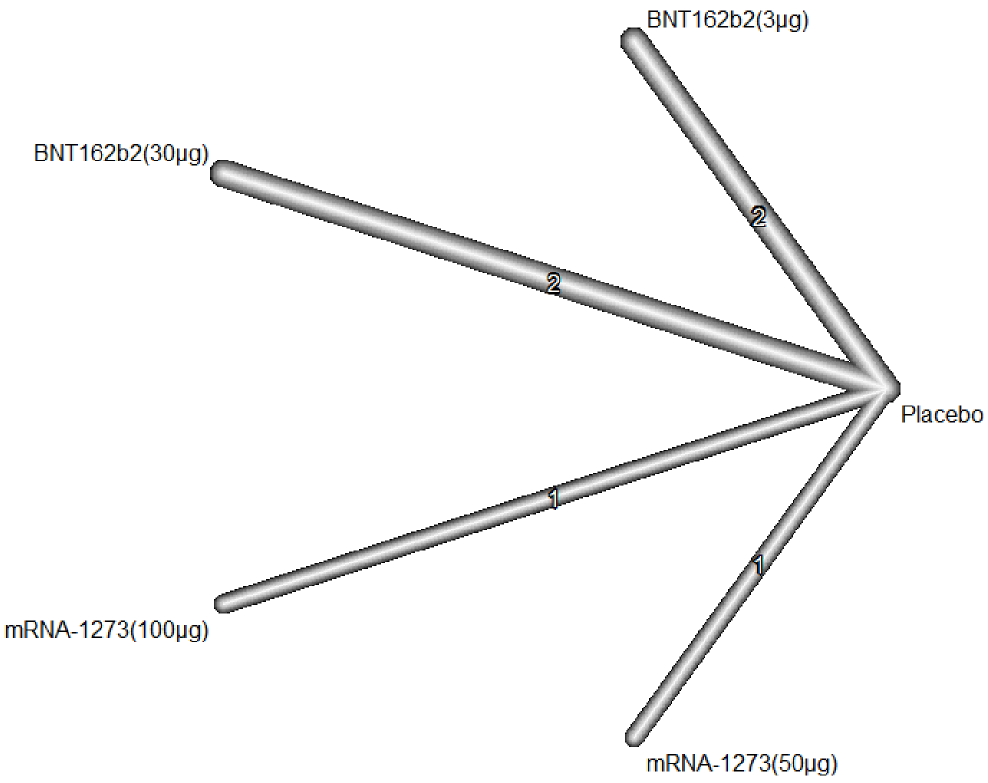

Figure S1: the network plot of the available COVID-19 vaccines that have been compared in phase 3 trials in children and adolescent.

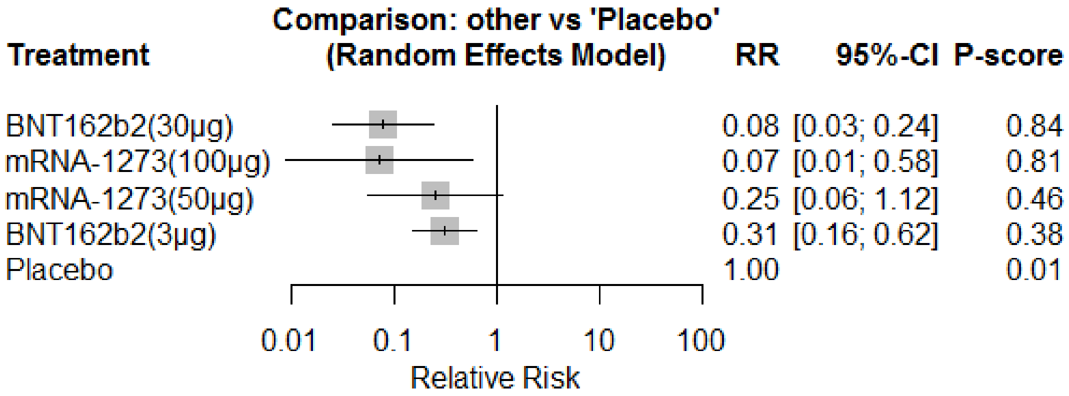

Figure S2: the forest plot for comparison of the available COVID-19 vaccines versus placebo in the network meta-analysis of RCTs in children and adolescent.

Table S1: the used search strategy in PubMed

#1: COVID-19 [Mesh Term]
#2: SARS-CoV-2[Mesh Term]
#3: #1 OR #2
#:4 COVID-19 Vaccines [Mesh Term]
#5: AstraZeneca [All field]
#6: ChAdOx1 nCoV-19 [Mesh Term]
#7: SINOPHARM [All field]
#8: SPUTNIK V [All field]
#9: Sputnik V COVID-19 vaccine [All field]
#10: Gam-COVID-Vac vaccine [Supplementary Concept]
#11: Pfizer [All field]
#12: BioNTech [All field]
#13: BNT162 Vaccine [Mesh Term]
#14 Johnson & Johnson [All field]
#15 Ad26COVS1 [Mesh Term]
#16: Moderna [All field]
#17 2019-nCoV Vaccine mRNA-1273 [Mesh Term]
#18: SINOVAC [All field]
#19: COVIran Barekat [All field]
#20: PastoCovac [All field]
#21: #4 OR #5 OR #6 OR …#21
#22: Effectiveness [All field]
#23: Vaccine Efficacy [Mesh Term]
#24: Mortality [Mesh Term]
#25: Complications [All field]
#26: Side effects [All field]
#27: adverse effects [Subheading] [Mesh Term]
#28 #22 OR #23 OR #24 OR … #27
#29 Randomized controlled trials [Mesh Terms]
#30 Clinical Trials as Topic [Mesh Terms]
#31 Clinical Trial [Publication Type]
#32 Controlled Clinical Trial [Publication Type]
#33: #29 OR #30 OR #31 OR #32
#34: #3 AND #21 AND #28 AND #33

Table S2: league table of the simultaneous comparison of available vaccines in term of preventing the symptomatic COVID-19 among adults

mRNA-1273(100µg)	0.9 (0.3, 2.7)	0.8 (0.2, 3.0)	0.5 (0.2, 1.5)	0.5 (0.1, 1.9)
.	BNT162b2(30µg)	0.9 (0.3, 2.8)	0.5 (0.2, 1.4)	0.5 (0.1, 1.9)
.	.	Gam-COVID-Vac	0.6 (0.2, 2.2)	0.6 (0.1, 2.7)
.	.	.	NVX-CoV2373	1.0 (0.3, 3.4)
.	.	.	.	CoronaVac
.	.	.	.	.
.	.	.	.	.
.	.	.	.	.
.	.	.	.	.
.	.	.	.	.
.	.	.	.	.
.	.	.	.	.
.	.	.	.	.
.	.	.	.	.
.	.	.	.	.
.	.	.	.	.
.	.	.	.	.
.	.	.	.	.
.	.	.	.	.
0.1 (0.0, 0.2)	0.1 (0.0, 0.1)	0.1 (0.0, 0.3)	0.1 (0.1, 0.3)	0.2 (0.1, 0.5)
.	.	.	.	.

0.2 (0.1, 0.7)	0.2 (0.1, 0.7)	0.2 (0.1, 0.8)	0.3 (0.1, 1.1)	0.4 (0.1, 1.3)	0.4 (0.1, 1.4)
0.2 (0.1, 0.6)	0.2 (0.1, 0.7)	0.2 (0.1, 0.8)	0.3 (0.1, 1.1)	0.4 (0.1, 1.2)	0.4 (0.1, 1.3)
0.2 (0.1, 1.0)	0.3 (0.1, 1.0)	0.3 (0.1, 1.1)	0.4 (0.1, 1.6)	0.5 (0.1, 1.8)	0.5 (0.1, 2.0)
0.4 (0.1, 1.2)	0.4 (0.1, 1.3)	0.4 (0.1, 1.4)	0.6 (0.2, 2.0)	0.8 (0.2, 2.3)	0.8 (0.3, 2.5)
0.4 (0.1, 1.7)	0.4 (0.1, 1.8)	0.5 (0.1, 2.0)	0.7 (0.2, 2.8)	0.8 (0.2, 3.2)	0.8 (0.2, 3.5)
0.5 (0.1, 1.8)	0.5 (0.1, 1.9)	0.5 (0.1, 2.1)	0.8 (0.2, 2.9)	0.9 (0.3, 3.4)	QazCovid-in(5μg)
0.5 (0.1, 1.8)	0.6 (0.2, 1.9)	0.6 (0.2, 2.1)	0.8 (0.2, 3.0)	ZF2001	.
0.6 (0.2, 2.2)	0.7 (0.2, 2.3)	0.7 (0.2, 2.6)	BBV152	.	.
0.9 (0.2, 3.3)	1.0 (0.3, 3.5)	ZyCoV-D(2mg)	.	.	.
0.9 (0.3, 3.2)	SCB-2019(30μg)	.	.	.	.
Soberana02+Soberana plus	.	.	.	.	.
.	.	.	.	.	.
.	.	.	.	.	.
.	.	.	.	.	.
.	.	.	.	.	.
.	.	.	.	.	.
.	.	.	.	.	.
.	.	.	.	.	.
.	.	.	.	.	.
0.4 (0.2, 0.9)	0.3 (0.1, 0.8)	0.3 (0.1, 0.9)	0.2 (0.1, 0.6)	0.2 (0.1, 0.5)	0.2 (0.1, 0.5)
.	.	.	.	.	.

0.1 (0.0, 0.4)	0.1 (0.0, 0.5)	0.2 (0.0, 0.6)	0.2 (0.1, 0.6)	0.2 (0.0, 0.8)	0.2 (0.1, 0.6)
0.1 (0.1, 0.3)	0.2 (0.1, 0.4)	0.2 (0.0, 0.6)	0.2 (0.1, 0.5)	0.2 (0.1, 0.8)	0.2 (0.1, 0.5)
0.2 (0.1, 0.6)	0.2 (0.0, 0.7)	0.2 (0.0, 0.9)	0.2 (0.1, 0.8)	0.2 (0.0, 1.1)	0.2 (0.1, 0.8)
0.3 (0.1, 0.7)	0.3 (0.1, 0.8)	0.3 (0.1, 1.1)	0.3 (0.1, 1.0)	0.4 (0.1, 1.5)	0.4 (0.2, 1.0)
0.3 (0.1, 1.0)	0.3 (0.1, 1.1)	0.3 (0.1, 1.5)	0.4 (0.1, 1.4)	0.4 (0.1, 2.0)	0.4 (0.1, 1.4)
0.3 (0.1, 1.0)	0.3 (0.1, 1.2)	0.4 (0.1, 1.6)	0.4 (0.1, 1.5)	0.5 (0.1, 2.1)	0.5 (0.2, 1.5)
0.4 (0.1, 1.0)	0.4 (0.1, 1.2)	0.4 (0.1, 1.6)	0.4 (0.1, 1.6)	0.5 (0.1, 2.2)	0.5 (0.2, 1.5)
0.4 (0.1, 1.3)	0.4 (0.1, 1.5)	0.5 (0.1, 2.0)	0.5 (0.1, 1.9)	0.6 (0.1, 2.6)	0.6 (0.2, 1.9)
0.6 (0.2, 1.9)	0.6 (0.2, 2.2)	0.7 (0.2, 2.9)	0.8 (0.2, 2.8)	0.8 (0.2, 3.9)	0.9 (0.3, 2.8)
0.6 (0.2, 1.8)	0.7 (0.2, 2.1)	0.7 (0.2, 2.9)	0.8 (0.2, 2.7)	0.9 (0.2, 3.8)	0.9 (0.3, 2.6)
0.7 (0.2, 2.0)	0.7 (0.2, 2.4)	0.8 (0.2, 3.1)	0.9 (0.2, 3.0)	0.9 (0.2, 4.2)	1.0 (0.3, 2.9)
0.7 (0.3, 1.6)	0.7 (0.3, 2.0)	0.8 (0.2, 2.8)	0.9 (0.3, 2.6)	1.0 (0.3, 3.7)	Ad26.COV2.S
0.7 (0.2, 2.7)	0.7 (0.2, 3.2)	0.8 (0.2, 4.1)	0.9 (0.2, 4.0)	Sinovac	.
0.8 (0.3, 2.3)	0.8 (0.2, 2.7)	0.9 (0.2, 3.6)	Ad5-nCoV	.	.
0.9 (0.3, 2.9)	0.9 (0.2, 3.4)	Ad5-nCoV(0.5mL)	.	.	.
1.0 (0.4, 2.7)	Soberana02	.	.	.	.
ChAdOx1 nCoV-19	.	.	.	.	.
.	.	.	.	.	.
.	.	.	.	.	.
0.5 (0.3, 1.0)	0.5 (0.2, 1.2)	0.5 (0.2, 1.3)	0.4 (0.2, 1.0)	0.4 (0.1, 1.3)	0.4 (0.2, 0.7)
0.3 (0.1, 0.7)	.	.	.	.	.

0.0 (0.0, 0.1)	0.1 (0.0, 0.2)	0.1 (0.0, 0.4)	0.1 (0.0, 0.4)
0.0 (0.0, 0.1)	0.1 (0.0, 0.1)	0.1 (0.0, 0.4)	0.1 (0.1, 0.4)
0.0 (0.0, 0.2)	0.1 (0.0, 0.3)	0.2 (0.0, 0.6)	0.2 (0.0, 0.6)
0.1 (0.0, 0.3)	0.1 (0.1, 0.3)	0.3 (0.1, 0.7)	0.3 (0.1, 0.8)
0.1 (0.0, 0.4)	0.2 (0.1, 0.5)	0.3 (0.1, 1.1)	0.3 (0.1, 1.1)
0.1 (0.0, 0.4)	0.2 (0.1, 0.5)	0.3 (0.1, 1.1)	0.3 (0.1, 1.1)
0.1 (0.0, 0.4)	0.2 (0.1, 0.5)	0.3 (0.1, 1.1)	0.3 (0.1, 1.2)
0.1 (0.0, 0.5)	0.2 (0.1, 0.6)	0.4 (0.1, 1.4)	0.4 (0.1, 1.4)
0.2 (0.0, 0.7)	0.3 (0.1, 0.9)	0.6 (0.2, 2.1)	0.6 (0.2, 2.1)
0.2 (0.0, 0.7)	0.3 (0.1, 0.8)	0.6 (0.2, 2.0)	0.6 (0.2, 2.0)
0.2 (0.0, 0.8)	0.4 (0.2, 0.9)	0.6 (0.2, 2.2)	0.7 (0.2, 2.2)
0.2 (0.1, 0.7)	0.4 (0.2, 0.7)	0.7 (0.2, 1.9)	0.7 (0.2, 1.9)
0.2 (0.0, 1.0)	0.4 (0.1, 1.3)	0.7 (0.2, 2.9)	0.7 (0.2, 3.0)
0.2 (0.1, 0.9)	0.4 (0.2, 1.0)	0.7 (0.2, 2.6)	0.8 (0.2, 2.6)
0.2 (0.1, 1.1)	0.5 (0.2, 1.3)	0.8 (0.2, 3.2)	0.8 (0.2, 3.2)
0.3 (0.1, 1.0)	0.5 (0.2, 1.2)	0.9 (0.3, 3.0)	0.9 (0.3, 3.0)
0.3 (0.1, 0.7)	0.5 (0.3, 1.0)	0.9 (0.3, 2.6)	1.0 (0.4, 2.7)
0.3 (0.1, 1.1)	0.6 (0.2, 1.2)	1.0 (0.3, 3.1)	BIV1-CovIran
0.3 (0.1, 1.2)	0.6 (0.2, 1.3)	CVnCoV	.
0.5 (0.2, 1.5)	Placebo	0.6 (0.2, 1.3)	0.6 (0.2, 1.2)
MenACWY	.	.	.

Table S3: league table of the simultaneous comparison of available vaccines in term of preventing the symptomatic COVID-19 among children and adolescent

BNT162b2(30µg)	.	.	.	0.1 (0.0, 0.2)
1.1 (0.1, 11.7)	mRNA-1273(100µg)	.	.	0.1 (0.0, 0.6)
0.3 (0.0, 2.0)	0.3 (0.0, 3.7)	mRNA-1273(50µg)	.	0.2 (0.1, 1.1)
0.2 (0.1, 0.9)	0.2 (0.0, 2.1)	0.8 (0.2, 4.2)	BNT162b2(3µg)	0.3 (0.2, 0.6)
0.1 (0.0, 0.2)	0.1 (0.0, 0.6)	0.2 (0.1, 1.1)	0.3 (0.2, 0.6)	Placebo

Table S4: league table of the simultaneous comparison of available vaccines in term of the incidence of local reaction among adults

0.2 (0.1, 0.4)	0.2 (0.1, 0.4)	0.2 (0.1, 0.4)	0.3 (0.2, 0.6)	0.5 (0.3, 0.9)	0.5 (0.2, 1.1)	0.5 (0.3, 0.9)	Gam-COVID-
0.4 (0.3, 0.5)	0.4 (0.3, 0.6)	0.4 (0.3, 0.5)	0.6 (0.4, 0.9)	0.9 (0.7, 1.3)	0.9 (0.5, 1.7)	Placebo	0.5 (0.3,0.9)
0.4 (0.2, 0.8)	0.4 (0.2, 0.8)	0.4 (0.2, 0.8)	0.6 (0.3, 1.2)	1.0 (0.5, 1.9)	ZyCoV-	0.9 (0.5,1.7)	.
0.4 (0.3, 0.7)	0.4 (0.3, 0.7)	0.4 (0.3, 0.7)	0.6 (0.4, 1.0)	BIV1-CovIran	.	0.9 (0.7,1.3)	.
0.7 (0.4, 1.1)	0.7 (0.4, 1.2)	0.7 (0.4, 1.2)	CoronaVac	.	.	0.6 (0.4,0.9)	.
0.9 (0.6, 1.5)	1.0 (0.6, 1.7)	Soberana02	.	.	.	0.4 (0.3,0.5)	.
0.9 (0.5, 1.6)	ChAdOx1	.	.	.	.	0.4 (0.3,0.6)	.
Soberana02+So	.	.	.	.	.	0.4 (0.3,0.5)	.
.	1.0 (0.7,1.4)	.	.	.	.	.	.
.	.	.	.	.	.	0.3 (0.3,0.4)	.
.	.	.	.	.	.	0.3 (0.2,0.5)	.
.	.	.	.	.	.	0.3 (0.2,0.5)	.
.	.	.	.	.	.	0.3 (0.2,0.4)	.
.	.	.	.	.	.	0.3 (0.2,0.5)	.
.	.	.	.	.	.	0.3 (0.2,0.4)	.
.	.	.	.	.	.	0.2 (0.2,0.3)	.
.	.	.	.	.	.	0.2 (0.1,0.3)	.
.	.	.	.	.	.	0.2 (0.1,0.2)	.
.	.	.	.	.	.	0.1 (0.1,0.2)	.
.	.	.	.	.	.	0.1 (0.0,0.2)	.

0.1 (0.1, 0.2)	0.1 (0.1, 0.2)	0.1 (0.1, 0.3)	0.1 (0.1, 0.3)	0.2 (0.1, 0.3)	0.2 (0.1, 0.3)	0.2 (0.1, 0.3)	0.2 (0.1, 0.3)	0.2 (0.1, 0.4)
0.2 (0.1, 0.3)	0.2 (0.2, 0.3)	0.3 (0.2, 0.4)	0.3 (0.2, 0.5)	0.3 (0.2, 0.4)	0.3 (0.2, 0.5)	0.3 (0.2, 0.5)	0.3 (0.3, 0.4)	0.4 (0.2, 0.7)
0.2 (0.1, 0.4)	0.2 (0.1, 0.4)	0.3 (0.1, 0.5)	0.3 (0.1, 0.6)	0.3 (0.2, 0.6)	0.3 (0.1, 0.6)	0.3 (0.2, 0.7)	0.3 (0.2, 0.6)	0.4 (0.2, 0.9)
0.2 (0.1, 0.3)	0.2 (0.1, 0.4)	0.3 (0.2, 0.4)	0.3 (0.2, 0.5)	0.3 (0.2, 0.5)	0.3 (0.2, 0.6)	0.3 (0.2, 0.5)	0.4 (0.2, 0.5)	0.4 (0.2, 0.8)
0.3 (0.2, 0.5)	0.4 (0.2, 0.6)	0.5 (0.3, 0.8)	0.5 (0.2, 0.9)	0.5 (0.3, 0.9)	0.5 (0.3, 1.0)	0.6 (0.3, 1.0)	0.6 (0.4, 0.9)	0.7 (0.3, 1.3)
0.5 (0.3, 0.7)	0.5 (0.3, 0.8)	0.7 (0.4, 1.0)	0.7 (0.4, 1.2)	0.7 (0.4, 1.2)	0.7 (0.4, 1.3)	0.8 (0.5, 1.3)	0.8 (0.6, 1.2)	1.0 (0.5, 1.8)
0.5 (0.3, 0.8)	0.5 (0.3, 0.9)	0.7 (0.4, 1.1)	0.7 (0.3, 1.3)	0.7 (0.4, 1.3)	0.7 (0.4, 1.4)	0.8 (0.5, 1.4)	0.8 (0.5, 1.3)	1.0 (0.7, 1.4)
0.5 (0.3, 0.8)	0.6 (0.3, 0.9)	0.7 (0.4, 1.1)	0.7 (0.4, 1.4)	0.8 (0.4, 1.3)	0.8 (0.4, 1.4)	0.9 (0.5, 1.4)	0.9 (0.6, 1.3)	1.0 (0.5, 2.0)
0.5 (0.2, 0.9)	0.6 (0.3, 1.0)	0.7 (0.4, 1.3)	0.7 (0.3, 1.5)	0.8 (0.4, 1.5)	0.8 (0.4, 1.6)	0.8 (0.4, 1.6)	0.9 (0.5, 1.6)	MVC-
0.6 (0.4, 0.8)	0.6 (0.4, 0.9)	0.8 (0.6, 1.2)	0.8 (0.5, 1.5)	0.9 (0.5, 1.4)	0.9 (0.5, 1.5)	1.0 (0.7, 1.5)	Ad26.COV2.S	.
0.6 (0.3, 0.9)	0.7 (0.4, 1.0)	0.8 (0.5, 1.3)	0.8 (0.4, 1.6)	0.9 (0.5, 1.5)	0.9 (0.5, 1.6)	Ad5-nCoV	.	.
0.6 (0.3, 1.1)	0.7 (0.4, 1.3)	0.9 (0.5, 1.7)	0.9 (0.4, 1.9)	1.0 (0.5, 1.9)	Sinovac	.	.	.
0.6 (0.4, 1.1)	0.7 (0.4, 1.2)	0.9 (0.5, 1.6)	0.9 (0.5, 1.8)	SCB-	.	.	.	.
0.7 (0.4, 1.3)	0.8 (0.4, 1.5)	1.0 (0.5, 1.9)	QazCovid-	.	.	.	.	.
0.7 (0.4, 1.1)	0.8 (0.5, 1.2)	NVX-	.	.	.	.	.	.
0.9 (0.5, 1.3)	mRNA-	.	.	.	.	.	.	.
CVnCoV	.	.	.	.	.	.	.	.
.	.	.	.	.	.	.	.	.
.	.	.	.	.	.	.	.	.
.	.	.	.	.	.	.	.	.

0.0 (0.0, 0.1)	0.1 (0.0, 0.1)	0.1 (0.0, 0.2)
0.1 (0.0, 0.2)	0.1 (0.1, 0.2)	0.2 (0.1, 0.2)
0.1 (0.0, 0.3)	0.1 (0.1, 0.2)	0.2 (0.1, 0.3)
0.1 (0.0, 0.3)	0.1 (0.1, 0.2)	0.2 (0.1, 0.3)
0.1 (0.0, 0.4)	0.2 (0.1, 0.4)	0.3 (0.2, 0.5)
0.1 (0.0, 0.6)	0.3 (0.2, 0.5)	0.4 (0.2, 0.6)
0.1 (0.0, 0.6)	0.3 (0.2, 0.5)	0.4 (0.2, 0.6)
0.1 (0.0, 0.6)	0.3 (0.2, 0.5)	0.4 (0.2, 0.7)
0.1 (0.0, 0.7)	0.3 (0.2, 0.6)	0.4 (0.2, 0.8)
0.2 (0.0, 0.7)	0.4 (0.3, 0.5)	0.5 (0.3, 0.7)
0.2 (0.0, 0.7)	0.4 (0.3, 0.6)	0.5 (0.3, 0.8)
0.2 (0.0, 0.9)	0.4 (0.3, 0.8)	0.5 (0.3, 1.0)
0.2 (0.0, 0.9)	0.4 (0.3, 0.7)	0.5 (0.3, 0.9)
0.2 (0.0, 1.0)	0.5 (0.3, 0.9)	0.6 (0.3, 1.1)
0.2 (0.0, 0.9)	0.5 (0.3, 0.7)	0.6 (0.4, 0.9)
0.3 (0.1, 1.1)	0.6 (0.4, 0.9)	0.7 (0.5, 1.1)
0.3 (0.1, 1.3)	0.7 (0.5, 1.1)	0.9 (0.5, 1.4)
0.4 (0.1, 1.6)	0.8 (0.5, 1.2)	BNT162b2
0.4 (0.1, 1.9)	BNT162b2(30	.
Ad5-	.	.

Table S5: league table of the simultaneous comparison of available vaccines in term of the incidence of fatigue among adults

0.2 (0.0, 1.2)	0.2 (0.0, 1.6)	0.2 (0.0, 1.7)	0.2 [0.0, 1.7)	0.2 [0.0, 1.7)	0.3 [0.0, 1.9)	0.4 [0.0, 3.6)	QazCovid-in(5μg)
0.4 (0.1, 1.2)	0.5 (0.2, 1.5)	0.5 (0.2, 1.7)	0.5 [0.2, 1.6)	0.5 [0.2, 1.6)	0.6 [0.3, 1.7)	ZyCoV-D(2mg)	.
0.6 (0.3, 1.0)	0.7 (0.4, 1.4)	0.8 (0.4, 1.5)	0.8 (0.5, 1.5)	0.8 [0.5, 1.5)	Placebo	0.6 [0.3, 1.7)	0.2 [0.0, 1.9)
0.7 (0.3, 1.6)	0.9 (0.4, 2.1)	1.0 (0.4, 2.3)	1.0 (0.4, 2.2)	CoronaVac	0.8 [0.5, 1.5)	.	.
0.7 (0.3, 1.6)	0.9 (0.4, 2.1)	1.0 (0.4, 2.3)	Soberana02	.	0.8 [0.5, 1.5)	.	.
0.7 (0.3, 1.7)	0.9 (0.4, 2.2)	Soberana02+Sobe rana plus	.	.	0.8 [0.4, 1.5)	.	.
0.8 (0.4, 1.9)	SCB-2019(30μg)	.	.	.	0.7 [0.4, 1.4)	.	.
ChAdOx1 nCoV-19	.	.	.	.	0.6 [0.3, 1.0)	.	.
.	.	.	.	.	0.6 [0.2, 2.0)	.	.
.	.	.	.	.	0.5 [0.4, 0.8)	.	.
0.9 (0.4, 1.7)	.	.	.	.	.	.	.
.	.	.	.	.	0.4 [0.2, 0.7)	.	.
.	.	.	.	.	0.4 [0.2, 0.7)	.	.
.	.	.	.	.	0.4 [0.2, 0.6)	.	.
.	.	.	.	.	0.3 [0.2, 0.6)	.	.
.	.	.	.	.	0.3 [0.2, 0.4)	.	.
.	.	.	.	.	0.2 [0.1, 0.4)	.	.

0.1 (0.0, 0.5)	0.1 (0.0, 0.7)	0.1 (0.0, 0.7)	0.1 (0.0, 0.8)	0.1 (0.0, 0.9)	0.1 (0.0, 1.2)	0.1 (0.0, 1.0)	0.1 (0.0, 1.6)
0.2 (0.1, 0.5)	0.2 (0.1, 0.7)	0.2 (0.1, 0.7)	0.2 (0.1, 0.7)	0.3 (0.1, 0.8)	0.3 (0.1, 1.2)	0.3 (0.1, 0.9)	0.4 (0.1, 1.8)
0.3 (0.2, 0.4)	0.3 (0.2, 0.6)	0.4 (0.2, 0.6)	0.4 (0.2, 0.7)	0.4 (0.2, 0.7)	0.5 (0.2, 1.2)	0.5 (0.4, 0.8)	0.6 (0.2, 2.0)
0.3 (0.2, 0.6)	0.4 (0.2, 0.9)	0.4 (0.2, 0.9)	0.4 (0.2, 1.0)	0.5 (0.2, 1.1)	0.6 (0.2, 1.8)	0.6 (0.3, 1.3)	0.7 (0.2, 2.7)
0.3 (0.2, 0.6)	0.4 (0.2, 0.9)	0.4 (0.2, 0.9)	0.4 (0.2, 1.0)	0.5 (0.2, 1.1)	0.6 (0.2, 1.8)	0.6 (0.3, 1.3)	0.7 (0.2, 2.7)
0.3 (0.1, 0.7)	0.4 (0.2, 1.0)	0.4 (0.2, 1.0)	0.4 (0.2, 1.0)	0.5 (0.2, 1.2)	0.6 (0.2, 1.8)	0.6 (0.3, 1.3)	0.7 (0.2, 2.7)
0.3 (0.2, 0.7)	0.5 (0.2, 1.1)	0.5 (0.2, 1.1)	0.5 (0.2, 1.2)	0.6 (0.2, 1.3)	0.7 (0.2, 2.1)	0.7 (0.3, 1.5)	0.8 (0.2, 3.1)
0.4 (0.2, 0.9)	0.6 (0.3, 1.3)	0.6 (0.3, 1.3)	0.6 (0.3, 1.4)	0.7 (0.3, 1.6)	0.9 (0.4, 1.7)	0.9 (0.4, 1.7)	1.0 (0.3, 3.7)
0.4 (0.1, 1.5)	0.6 (0.2, 2.2)	0.6 (0.2, 2.2)	0.6 (0.2, 2.3)	0.7 (0.2, 2.7)	0.9 (0.2, 3.8)	0.9 (0.3, 3.1)	Ad5-nCoV(0.5mL)
0.5 (0.3, 0.9)	0.7 (0.3, 1.3)	0.7 (0.3, 1.3)	0.7 (0.4, 1.4)	0.8 (0.4, 1.6)	1.0 (0.4, 2.6)	Ad26.COV2.S	.
0.5 (0.2, 1.3)	0.7 (0.2, 1.9)	0.7 (0.2, 1.9)	0.7 (0.3, 2.0)	0.8 (0.3, 2.3)	MVC-COV1901	.	.
0.6 (0.3, 1.2)	0.8 (0.4, 1.8)	0.8 (0.4, 1.8)	0.9 (0.4, 1.9)	NVX-CoV2373	.	.	.
0.7 (0.3, 1.4)	0.9 (0.4, 2.1)	1.0 (0.4, 2.1)	CVnCoV	.	.	.	.
0.7 (0.4, 1.4)	1.0 (0.4, 2.2)	mRNA-1273(100µg)	.	.	.	.	.
0.7 (0.4, 1.5)	BNT162b2	.	.	.	.	.	.
BNT162b2(30µg)	.	.	.	.	.	.	.
.	.	.	.	.	.	.	.

0.0 (0.0, 0.4)
0.1 (0.0, 0.4)
0.2 (0.1, 0.4)
0.2 (0.1, 0.6)
0.2 (0.1, 0.6)
0.2 (0.1, 0.6)
0.2 (0.1, 0.7)
0.3 (0.1, 0.8)
0.3 (0.1, 1.2)
0.3 (0.1, 0.9)
0.3 (0.1, 1.1)
0.4 (0.1, 1.1)
0.4 (0.1, 1.3)
0.4 (0.1, 1.4)
0.5 (0.1, 1.4)
0.6 (0.2, 1.8)
Sinovac

Table S6: league table of the simultaneous comparison of available vaccines in term of the incidence of chill among adults

0.1 (0.0, 2.8)	0.2 (0.0, 2.2)	0.2 (0.0, 3.3)	0.3 (0.0, 5.7)	0.3 (0.0, 4.6)	0.4 (0.0, 5.6)	0.4 (0.0, 3.2)	QazCovid-in(5µg)
0.3 (0.0, 3.3)	0.4 (0.1, 1.9)	0.5 (0.1, 3.3)	0.8 (0.1, 6.1)	0.8 (0.1, 4.5)	1.0 (0.2, 5.5)	Placebo	0.4 (0.0,3.2)
0.3 (0.0, 6.1)	0.4 (0.0, 4.5)	0.5 (0.0, 7.0)	0.8 (0.1,12.2)	0.8 (0.1, 9.7)	Soberana02	1.0 (0.2,5.5)	.
0.4 (0.0, 7.4)	0.5 (0.1, 5.4)	0.7 (0.1, 8.5)	1.0 (0.1,14.8)	CoronaVac	.	0.8 (0.1,4.5)	.
0.4 (0.0, 8.7)	0.5 (0.0, 6.6)	0.7 (0.0,10.2)	Soberana02+Soberana plus	.	.	0.8 (0.1,6.1)	.
0.6 (0.0,12.0)	0.8 (0.1, 8.8)	SCB-2019(30µg)	.	.	.	0.5 (0.1,3.3)	.
0.7 (0.1, 4.6)	ChAdOx1 nCoV-19	.	.	.	.	0.4 (0.1,1.9)	.
MVC-COV1901	0.7 (0.1,4.6)	.	.	.	.	.	.
.	.	.	.	.	.	0.1 (0.0,0.7)	.
.	.	.	.	.	.	0.1 (0.0,0.6)	.
.	.	.	.	.	.	0.1 (0.0,0.6)	.
.	.	.	.	.	.	0.1 (0.0,0.3)	.

0.0 (0.0, 0.3)	0.0 (0.0, 0.6)	0.0 (0.0, 0.6)	0.0 (0.0, 0.7)
0.1 (0.0, 0.3)	0.1 (0.0, 0.6)	0.1 (0.0, 0.6)	0.1 (0.0, 0.7)
0.1 (0.0, 0.7)	0.1 (0.0, 1.2)	0.1 (0.0, 1.2)	0.1 (0.0, 1.5)
0.1 (0.0, 0.8)	0.1 (0.0, 1.5)	0.1 (0.0, 1.5)	0.2 (0.0, 1.8)
0.1 (0.0, 1.0)	0.1 (0.0, 1.8)	0.1 (0.0, 1.8)	0.2 (0.0, 2.2)
0.1 (0.0, 1.3)	0.2 (0.0, 2.4)	0.2 (0.0, 2.5)	0.2 (0.0, 3.0)
0.2 (0.0, 1.3)	0.2 (0.0, 2.5)	0.2 (0.0, 2.5)	0.3 (0.0, 3.1)
0.2 (0.0, 3.7)	0.3 (0.0, 6.5)	0.3 (0.0, 6.6)	0.4 (0.0, 8.1)
0.6 (0.1, 4.9)	0.8 (0.1, 9.1)	0.8 (0.1, 9.3)	mRNA-1273(100µg)
0.7 (0.1, 6.1)	1.0 (0.1,11.4)	CVnCoV	.
0.7 (0.1, 6.2)	BNT162b2	.	.
BNT162b2(30µg)	.	.	.

Table S7: league table of the simultaneous comparison of available vaccines in term of the incidence of fever among adults

0.2 (0.0, 1.2)	0.3 (0.1, 0.7)	0.2 (0.0, 1.6)	0.4 (0.0, 3.7)	0.4 (0.2, 0.9)	0.4 (0.1, 1.2)	0.4 (0.2, 1.0)	0.5 (0.2, 1.1)	QazCovid-in(5µg)
0.4 (0.1, 2.1)	0.6 (0.4, 0.9)	0.5 (0.1, 2.7)	0.8 (0.1, 6.7)	0.8 (0.6, 1.1)	0.8 (0.4, 1.7)	0.9 (0.7, 1.0)	Placebo	0.5 (0.2, 1.1)
0.5 (0.1, 2.4)	0.7 (0.4, 1.1)	0.6 (0.1, 3.2)	0.9 (0.1, 7.8)	0.9 (0.7, 1.3)	0.9 (0.4, 2.0)	Soberana02	0.9 (0.7, 1.0)	.
0.5 (0.1, 3.0)	0.8 (0.3, 1.8)	0.6 (0.1, 3.9)	0.9 (0.1, 9.3)	1.0 (0.5, 2.2)	ZyCoV-D(2mg)	.	0.8 (0.4, 1.7)	.
0.5 (0.1, 2.6)	0.7 (0.4, 1.3)	0.6 (0.1, 3.4)	0.9 (0.1, 8.4)	CoronaVac	.	.	0.8 (0.6, 1.1)	.
0.5 (0.0, 8.2)	0.8 (0.1, 7.6)	0.7 (0.0, 10.6)	Gam-COVID-Vac	.	.	.	0.8 (0.1, 6.7)	.
0.8 (0.1, 8.4)	1.2 (0.2, 7.0)	Sinovac	.	.	.	.	0.5 (0.1, 2.7)	.
0.7 (0.1, 3.6)	Soberana02+Soberana plus	.	.	.	.	.	0.6 (0.4, 0.9)	.
SCB-2019(30µg)	.	.	.	.	.	.	0.4 (0.1, 2.1)	.
.	.	.	.	.	.	.	0.3 (0.2, 0.6)	.
.	.	.	.	.	.	.	0.1 (0.1, 0.2)	.
.	.	.	.	.	.	.	0.0 (0.0, 0.1)	.
.	.	.	.	.	.	.	0.0 (0.0, 0.0)	.
.	.	.	.	.	.	.	0.0 (0.0, 0.0)	.
.	.	.	.	.	.	.	0.0 (0.0, 0.0)	.
.	.	.	.	.	.	.	0.0 (0.0, 0.0)	.
.	.	.	.	.	.	.	0.0 (0.0, 0.0)	.

0.0 (0.0, 0.0)	0.0 (0.0, 0.0)	0.0 (0.0, 0.0)	0.0 (0.0, 0.0)	0.0 (0.0, 0.0)	0.0 (0.0, 0.1)	0.1 (0.0, 0.2)	0.1 (0.0, 0.4)
0.0 (0.0, 0.0)	0.0 (0.0, 0.0)	0.0 (0.0, 0.0)	0.0 (0.0, 0.0)	0.0 (0.0, 0.0)	0.0 (0.0, 0.1)	0.1 (0.1, 0.2)	0.3 (0.2, 0.6)
0.0 (0.0, 0.0)	0.0 (0.0, 0.0)	0.0 (0.0, 0.0)	0.0 (0.0, 0.0)	0.0 (0.0, 0.0)	0.1 (0.0, 0.1)	0.1 (0.1, 0.2)	0.4 (0.2, 0.8)
0.0 (0.0, 0.0)	0.0 (0.0, 0.0)	0.0 (0.0, 0.0)	0.0 (0.0, 0.0)	0.0 (0.0, 0.1)	0.1 (0.0, 0.1)	0.2 (0.1, 0.4)	0.4 (0.1, 1.1)
0.0 (0.0, 0.0)	0.0 (0.0, 0.0)	0.0 (0.0, 0.0)	0.0 (0.0, 0.0)	0.0 (0.0, 0.0)	0.1 (0.0, 0.1)	0.2 (0.1, 0.3)	0.4 (0.2, 0.8)
0.0 (0.0, 0.0)	0.0 (0.0, 0.0)	0.0 (0.0, 0.1)	0.0 (0.0, 0.2)	0.0 (0.0, 0.2)	0.1 (0.0, 0.6)	0.2 (0.0, 1.6)	0.4 (0.0, 4.2)
0.0 (0.0, 0.0)	0.0 (0.0, 0.0)	0.0 (0.0, 0.1)	0.0 (0.0, 0.1)	0.0 (0.0, 0.2)	0.1 (0.0, 0.5)	0.3 (0.0, 1.4)	0.6 (0.1, 4.0)
0.0 (0.0, 0.0)	0.0 (0.0, 0.0)	0.0 (0.0, 0.0)	0.0 (0.0, 0.0)	0.0 (0.0, 0.1)	0.1 (0.0, 0.1)	0.2 (0.1, 0.4)	0.5 (0.2, 1.2)
0.0 (0.0, 0.0)	0.0 (0.0, 0.0)	0.0 (0.0, 0.1)	0.0 (0.0, 0.2)	0.0 (0.0, 0.3)	0.1 (0.0, 0.6)	0.3 (0.1, 1.7)	0.8 (0.1, 4.7)
0.0 (0.0, 0.0)	0.0 (0.0, 0.0)	0.0 (0.0, 0.1)	0.0 (0.0, 0.1)	0.1 (0.0, 0.1)	0.1 (0.1, 0.3)	0.4 (0.2, 0.9)	Ad5-nCoV(0·5mL)
0.0 (0.0, 0.0)	0.0 (0.0, 0.0)	0.0 (0.0, 0.2)	0.1 (0.0, 0.2)	0.1 (0.1, 0.2)	0.4 (0.2, 0.7)	Ad5-nCoV	.
0.0 (0.0, 0.1)	0.0 (0.0, 0.1)	0.0 (0.0, 0.5)	0.2 (0.1, 0.6)	0.4 (0.2, 0.7)	NVX-CoV2373	.	.
0.0 (0.0, 0.1)	0.0 (0.0, 0.2)	0.1 (0.0, 1.3)	0.6 (0.2, 1.5)	mRNA-1273(100µg)	.	.	.
0.0 (0.0, 0.3)	0.0 (0.0, 0.4)	0.1 (0.0, 2.6)	CVnCoV	.	.	.	.
0.1 (0.0, 4.9)	0.1 (0.0, 7.5)	BNT162b2	.	.	.	.	.
0.7 (0.0, 33.3)	Ad26.COV2.S	.	.	.	.	.	.
BNT162b2(30µg)	.	.	.	.	.	.	.

Supplementary Table 8: league table of the simultaneous comparison of available vaccines in term of the incidence of pain among adults

0.3 (0.1, 0.6)	0.3 (0.1, 0.6)	0.3 (0.1, 0.7)	0.3 (0.1, 0.9)	0.4 (0.1, 2.6)	0.3 (0.2, 0.7)	1.1 (0.2, 5.1)	Ad5-nCoV(0.5mL)
0.2 (0.1, 1.0)	0.2 (0.1, 1.0)	0.3 (0.1, 1.1)	0.3 (0.1, 1.4)	0.4 (0.0, 3.3)	0.3 (0.1, 1.2)	QazCovid-in(5μg)	.
0.8 (0.5, 1.2)	0.8 (0.5, 1.2)	0.8 (0.5, 1.5)	0.9 (0.4, 2.0)	1.2 (0.2, 6.5)	Placebo	0.3 (0.1; 1.2)	0.3 (0.2; 0.7)
0.6 (0.1, 3.6)	0.6 (0.1, 3.7)	0.7 (0.1, 4.1)	0.7 (0.1, 4.8)	Gam-COVID-Vac	1.2 (0.2; 6.5)	.	.
0.9 (0.3, 2.4)	0.9 (0.3, 2.4)	1.0 (0.3, 2.7)	ZyCoV-D(2mg)	.	0.9 (0.4; 2.0)	.	.
0.9 (0.4, 1.9)	0.9 (0.5, 1.9)	SCB-2019(30μg)	.	.	0.8 (0.5; 1.5)	.	.
1.0 (0.5, 1.9)	Soberana02	.	.	.	0.8 (0.5; 1.2)	.	.
CoronaVac	.	.	.	.	0.8 (0.5; 1.2)	.	.
.	.	.	.	.	0.5 (0.3; 0.7)	.	.
.	.	.	.	.	0.4 (0.2; 0.9)	.	.
.	.	.	.	.	0.4 (0.3; 0.5)	.	.
.	.	.	.	.	0.3 (0.2; 0.4)	.	.
.	.	.	.	.	0.2 (0.2; 0.4)	.	.
.	.	.	.	.	0.2 (0.2; 0.4)	.	.
.	.	.	.	.	0.2 (0.1; 0.3)	.	.
.	.	.	.	.	.	.	.
.	.	.	.	.	0.2 (0.1; 0.3)	.	.
.	.	.	.	.	0.2 (0.1; 0.2)	.	.
.	.	.	.	.	0.1 (0.0; 0.3)	.	.

0.1 (0.0, 0.1)	0.1 (0.0, 0.2)	0.1 (0.0, 0.2)	0.1 (0.0, 0.2)	0.1 (0.0, 0.2)	0.1 (0.0, 0.2)	0.1 (0.1, 0.3)	0.1 (0.0, 0.4)	0.2 (0.1, 0.4)
0.1 (0.0, 0.2)	0.1 (0.0, 0.3)	0.1 (0.0, 0.3)	0.1 (0.0, 0.3)	0.1 (0.0, 0.3)	0.1 (0.0, 0.4)	0.1 (0.0, 0.5)	0.1 (0.0, 0.6)	0.1 (0.0, 0.6)
0.2 (0.1, 0.3)	0.2 (0.1, 0.4)	0.2 (0.1, 0.3)	0.2 (0.2, 0.4)	0.2 (0.2, 0.4)	0.3 (0.2, 0.4)	0.4 (0.3, 0.5)	0.4 (0.2, 0.9)	0.5 (0.3, 0.7)
0.1 (0.0, 0.8)	0.2 (0.0, 1.1)	0.2 (0.0, 1.0)	0.2 (0.0, 1.1)	0.2 (0.0, 1.2)	0.2 (0.0, 1.3)	0.3 (0.1, 1.7)	0.3 (0.1, 2.2)	0.4 (0.1, 2.2)
0.2 (0.1, 0.5)	0.2 (0.1, 0.8)	0.2 (0.1, 0.7)	0.3 (0.1, 0.7)	0.3 (0.1, 0.8)	0.3 (0.1, 0.9)	0.4 (0.2, 1.1)	0.5 (0.2, 1.5)	0.5 (0.2, 1.4)
0.2 (0.1, 0.4)	0.3 (0.1, 0.6)	0.3 (0.1, 0.5)	0.3 (0.1, 0.6)	0.3 (0.1, 0.6)	0.3 (0.2, 0.7)	0.4 (0.2, 0.9)	0.5 (0.2, 1.3)	0.6 (0.3, 1.2)
0.2 (0.1, 0.4)	0.3 (0.1, 0.6)	0.3 (0.2, 0.5)	0.3 (0.2, 0.6)	0.3 (0.2, 0.6)	0.4 (0.2, 0.7)	0.5 (0.3, 0.8)	0.5 (0.2, 1.3)	0.6 (0.3, 1.1)
0.2 (0.1, 0.4)	0.3 (0.1, 0.6)	0.3 (0.2, 0.5)	0.3 (0.2, 0.6)	0.3 (0.2, 0.6)	0.4 (0.2, 0.7)	0.5 (0.3, 0.8)	0.6 (0.2, 1.3)	0.6 (0.3, 1.2)
0.4 (0.2, 0.7)	0.5 (0.2, 1.0)	0.5 (0.3, 0.8)	0.5 (0.3, 0.9)	0.5 (0.3, 1.0)	0.6 (0.3, 1.1)	0.8 (0.5, 1.3)	0.9 (0.4, 2.1)	Ad5-nCoV
0.4 (0.2, 1.0)	0.5 (0.2, 1.4)	0.5 (0.2, 1.2)	0.6 (0.2, 1.3)	0.6 (0.2, 1.4)	0.7 (0.3, 1.5)	0.9 (0.4, 2.0)	Soberana02+Soberana plus	.
0.5 (0.3, 0.8)	0.6 (0.3, 1.2)	0.6 (0.3, 1.0)	0.7 (0.4, 1.1)	0.7 (0.4, 1.1)	0.7 (0.4, 1.3)	Ad26.COV2.S	.	.
0.6 (0.3, 1.1)	0.8 (0.4, 1.3)	0.8 (0.4, 1.4)	0.9 (0.5, 1.6)	0.9 (0.5, 1.6)	ChAdOx1 nCoV-19	.	.	.
0.7 (0.4, 1.3)	0.9 (0.4, 2.0)	0.9 (0.5, 1.6)	1.0 (0.5, 1.8)	BNT162b2	.	.	.	.
0.7 (0.4, 1.3)	0.9 (0.4, 2.0)	0.9 (0.5, 1.6)	NVX-CoV2373	.	.	.	.	.
0.8 (0.4, 1.4)	1.0 (0.4, 2.2)	mRNA-1273(100μg)	.	.	.	.	.	.
0.8 (0.3, 1.8)	MVC-COV1901	.	.	.	0.8 (0.4; 1.3)	.	.	.
CVnCoV	.	.	.	.	.	.	.	.
.	.	.	.	.	.	.	.	.
.	.	.	.	.	.	.	.	.

0.0 (0.0, 0.1)	0.1 (0.0, 0.1)
0.0 (0.0, 0.2)	0.0 (0.0, 0.2)
0.1 (0.0, 0.3)	0.2 (0.1, 0.2)
0.1 (0.0, 0.6)	0.1 (0.0, 0.7)
0.1 (0.0, 0.4)	0.2 (0.1, 0.5)
0.1 (0.0, 0.4)	0.2 (0.1, 0.4)
0.1 (0.1, 0.4)	0.2 (0.1, 0.3)
0.1 (0.1, 0.4)	0.2 (0.1, 0.4)
0.2 (0.1, 0.6)	0.3 (0.2, 0.6)
0.3 (0.1, 0.8)	0.4 (0.2, 0.8)
0.3 (0.1, 0.7)	0.4 (0.3, 0.6)
0.4 (0.2, 1.0)	0.6 (0.3, 0.9)
0.5 (0.2, 1.2)	0.6 (0.4, 1.1)
0.5 (0.2, 1.2)	0.6 (0.4, 1.1)
0.5 (0.2, 1.3)	0.7 (0.4, 1.2)
0.5 (0.2, 1.6)	0.7 (0.3, 1.6)
0.7 (0.3, 1.7)	0.9 (0.5, 1.6)
0.7 (0.3, 1.8)	BNT162b2(30µg)
Sinovac	.

Table S9: league table of the simultaneous comparison of available vaccines in term of the incidence of headache among adults

0.6 (0.2, 2.2)	0.7 (0.2, 2.2)	0.7 (0.3, 1.8)	0.7 (0.3, 1.8)	0.7 (0.3, 1.8)	0.8 (0.3, 2.3)	0.8 (0.3, 1.6)	0.8 (0.3, 2.0)	QazCovid-in(5µg)
0.8 (0.3, 2.4)	0.9 (0.3, 2.4)	0.9 (0.4, 1.9)	0.9 (0.4, 1.9)	0.9 (0.4, 1.9)	1.0 (0.4, 2.6)	1.0 (0.6, 1.6)	CoronaVac	.
0.8 (0.3, 2.2)	0.9 (0.4, 2.2)	0.9 (0.5, 1.6)	0.9 (0.5, 1.6)	0.9 (0.6, 1.6)	1.0 (0.5, 2.3)	Placebo	1.0 (0.6; 1.6)	0.8 (0.3; 1.6)
0.8 (0.2, 2.8)	0.9 (0.3, 2.8)	0.9 (0.3, 2.3)	0.9 (0.3, 2.3)	0.9 (0.4, 2.3)	ZyCoV-D(2mg)	1.0 (0.5; 2.3)	.	.
0.9 (0.3, 2.7)	1.0 (0.3, 2.7)	1.0 (0.4, 2.1)	1.0 (0.5, 2.1)	Soberana02	.	0.9 (0.6; 1.6)	.	.
0.9 (0.3, 2.8)	1.0 (0.3, 2.9)	1.0 (0.4, 2.2)	SCB-2019(30µg)	.	.	0.9 (0.5; 1.6)	.	.
0.9 (0.3, 2.9)	1.0 (0.3, 2.9)	Soberana02+Soberana plus	.	.	.	0.9 (0.5; 1.6)	.	.
0.9 (0.2, 3.4)	Gam-COVID-Vac	.	.	.	.	0.9 (0.4; 2.2)	.	.
Ad5-nCoV(0.5mL)	.	.	.	.	.	0.8 (0.3; 2.2)	.	.
.	.	.	.	.	.	0.7 (0.4; 1.1)	.	.
.	.	.	.	.	.	.	.	.
.	.	.	.	.	.	0.6 (0.3; 0.9)	.	.
.	.	.	.	.	.	0.5 (0.4; 0.8)	.	.
.	.	.	.	.	.	0.4 (0.3; 0.7)	.	.
.	.	.	.	.	.	0.4 (0.2; 0.7)	.	.
.	.	.	.	.	.	0.4 (0.2; 0.6)	.	.
.	.	.	.	.	.	0.3 (0.2; 0.5)	.	.
.	.	.	.	.	.	0.3 (0.2; 0.4)	.	.

0.2 (0.1, 0.5)	0.2 (0.1, 0.6)	0.3 (0.1, 0.7)	0.3 (0.1, 0.7)	0.3 (0.1, 0.8)	0.4 (0.2, 0.9)	0.4 (0.2, 1.0)	0.4 (0.1, 1.2)	0.5 (0.2, 1.3)
0.3 (0.2, 0.5)	0.3 (0.2, 0.6)	0.4 (0.2, 0.8)	0.4 (0.2, 0.8)	0.4 (0.2, 0.8)	0.5 (0.3, 1.0)	0.5 (0.3, 1.1)	0.5 (0.2, 1.4)	0.7 (0.3, 1.4)
0.3 (0.2, 0.4)	0.3 (0.2, 0.5)	0.4 (0.2, 0.6)	0.4 (0.2, 0.7)	0.4 (0.3, 0.7)	0.5 (0.4, 0.8)	0.6 (0.3, 0.9)	0.6 (0.3, 1.2)	0.7 (0.4, 1.1)
0.3 (0.1, 0.7)	0.3 (0.1, 0.8)	0.4 (0.1, 0.9)	0.4 (0.2, 0.9)	0.4 (0.2, 1.0)	0.5 (0.2, 1.2)	0.5 (0.2, 1.3)	0.5 (0.2, 1.6)	0.7 (0.3, 1.7)
0.3 (0.2, 0.6)	0.3 (0.2, 0.7)	0.4 (0.2, 0.9)	0.4 (0.2, 0.9)	0.5 (0.2, 0.9)	0.6 (0.3, 1.1)	0.6 (0.3, 1.2)	0.6 (0.2, 1.5)	0.7 (0.4, 1.5)
0.3 (0.2, 0.6)	0.4 (0.2, 0.8)	0.4 (0.2, 0.9)	0.4 (0.2, 0.9)	0.5 (0.2, 1.0)	0.6 (0.3, 1.2)	0.6 (0.3, 1.3)	0.6 (0.2, 1.6)	0.8 (0.4, 1.6)
0.3 (0.2, 0.7)	0.4 (0.2, 0.8)	0.4 (0.2, 0.9)	0.4 (0.2, 0.9)	0.5 (0.2, 1.0)	0.6 (0.3, 1.2)	0.6 (0.3, 1.3)	0.6 (0.2, 1.6)	0.8 (0.4, 1.6)
0.3 (0.1, 0.9)	0.4 (0.1, 1.0)	0.4 (0.2, 1.2)	0.4 (0.2, 1.2)	0.5 (0.2, 1.3)	0.6 (0.2, 1.6)	0.6 (0.2, 1.7)	0.6 (0.2, 2.0)	0.8 (0.3, 2.1)
0.4 (0.1, 1.1)	0.4 (0.1, 1.2)	0.5 (0.2, 1.5)	0.5 (0.2, 1.5)	0.5 (0.2, 1.6)	0.7 (0.2, 1.9)	0.7 (0.2, 2.1)	0.7 (0.2, 2.4)	0.9 (0.3, 2.6)
0.4 (0.2, 0.8)	0.5 (0.2, 1.0)	0.6 (0.3, 1.2)	0.6 (0.3, 1.2)	0.6 (0.3, 1.3)	0.8 (0.4, 1.5)	0.8 (0.4, 1.6)	0.8 (0.3, 2.0)	Ad5-nCoV
0.5 (0.2, 1.3)	0.6 (0.2, 1.5)	0.7 (0.3, 1.8)	0.7 (0.3, 1.8)	0.8 (0.3, 1.9)	1.0 (0.4, 2.3)	1.0 (0.5, 1.8)	MVC-COV1901	.
0.5 (0.3, 1.0)	0.6 (0.3, 1.2)	0.7 (0.4, 1.4)	0.7 (0.4, 1.4)	0.8 (0.4, 1.5)	1.0 (0.5, 1.8)	ChAdOx1 nCoV-[illegible]	1.0 (0.5; 1.8)	.
0.5 (0.3, 0.9)	0.6 (0.3, 1.1)	0.7 (0.4, 1.3)	0.7 (0.4, 1.4)	0.8 (0.4, 1.5)	Ad26.COV2.S	.	.	.
0.7 (0.4, 1.3)	0.8 (0.4, 1.5)	0.9 (0.5, 1.9)	0.9 (0.5, 1.9)	NVX-CoV2373	.	.	.	.
0.7 (0.4, 1.4)	0.8 (0.4, 1.7)	1.0 (0.5, 2.0)	mRNA-[illegible]	.	.	.	.	.
0.8 (0.4, 1.4)	0.8 (0.4, 1.7)	BNT162b2	.	.	.	.	.	.
0.9 (0.5, 1.7)	CVnCoV	.	.	.	.	.	.	.
BNT162b2(30µg[illegible]	.	.	.	.	.	.	.	.
